# Crystal structures of (±)-(1*SR*,5*SR*,6*SR*,7*SR*,10*SR*,11*SR*,13*RS*,14*SR*,15*SR*,16*RS*)-13-acet­oxy-16-benzyloxy-15-hy­droxy-7-meth­oxy­meth­oxy-3-oxo-11,15,18,18-tetra­methyl-2,4-dioxa­tetra­cyclo[12.3.1.0^1,5^.0^6,11^]octa­decan-10-yl benzoate and its 13-epimer

**DOI:** 10.1107/S2056989024012234

**Published:** 2025-01-01

**Authors:** Takeshi Oishi, Keisuke Fukaya, Takaaki Sato, Noritaka Chida

**Affiliations:** a School of Medicine, Keio University, Hiyoshi 4-1-1, Kohoku-ku, Yokohama 223-8521, Japan; b Department of Applied Chemistry, Faculty of Science and Technology, Keio University, Hiyoshi 3-14-1, Kohoku-ku, Yokohama 223-8522, Japan; University of Hyogo, Japan

**Keywords:** crystal structure, hydrogen bond, taxane skeleton, paclitaxel, disorder

## Abstract

The title compounds, C_38_H_48_O_11_ (**A** and **B**), are tetra­cyclic benzoates composed of a taxane ring with a fused dioxolane ring as the core skeleton. In compound **A**, the five-membered dioxolane ring is essentially planar while the two cyclo­hexane rings and the cyclo­octane ring adopt chair and chair–chair forms, respectively, and there are three intra­molecular H⋯H short contacts. The corresponding ring conformations in **B** are similar; however, one intra­molecular C—H⋯O inter­action and two H⋯H short contacts are observed, and the benzoyl and meth­oxy­methyl groups show orientational disorder. In the crystal of **A**, a pair of inter­molecular O—H⋯O hydrogen bonds link two mol­ecules into an inversion dimer, and weak inter­molecular C—H⋯O inter­actions connect the dimers, forming a three-dimensional network. In the crystal of **B**, an inversion dimer is similarly generated by a pair of inter­molecular O—H⋯O hydrogen bonds, and weak inter­molecular C—H⋯O and C—H⋯π inter­actions connect the dimers into a three-dimensional architecture.

## Abstract 2

The crystal structures of a fused tetra­cyclic benzoate and its epimer obtained in synthetic study of Paclitaxel are described. The corresponding ring conformations in both tetra­cycles are similar; however, one epimer shows two orientational disorders and while none occurs for the other. In each crystal, pairs of inter­molecular O—H⋯O hydrogen bonds connect the mol­ecules into inversion dimers. The dimers are further linked by weak inter­molecular C—H⋯O and/or C—H⋯π inter­actions.

## 1. Chemical context

**Figure 1:**
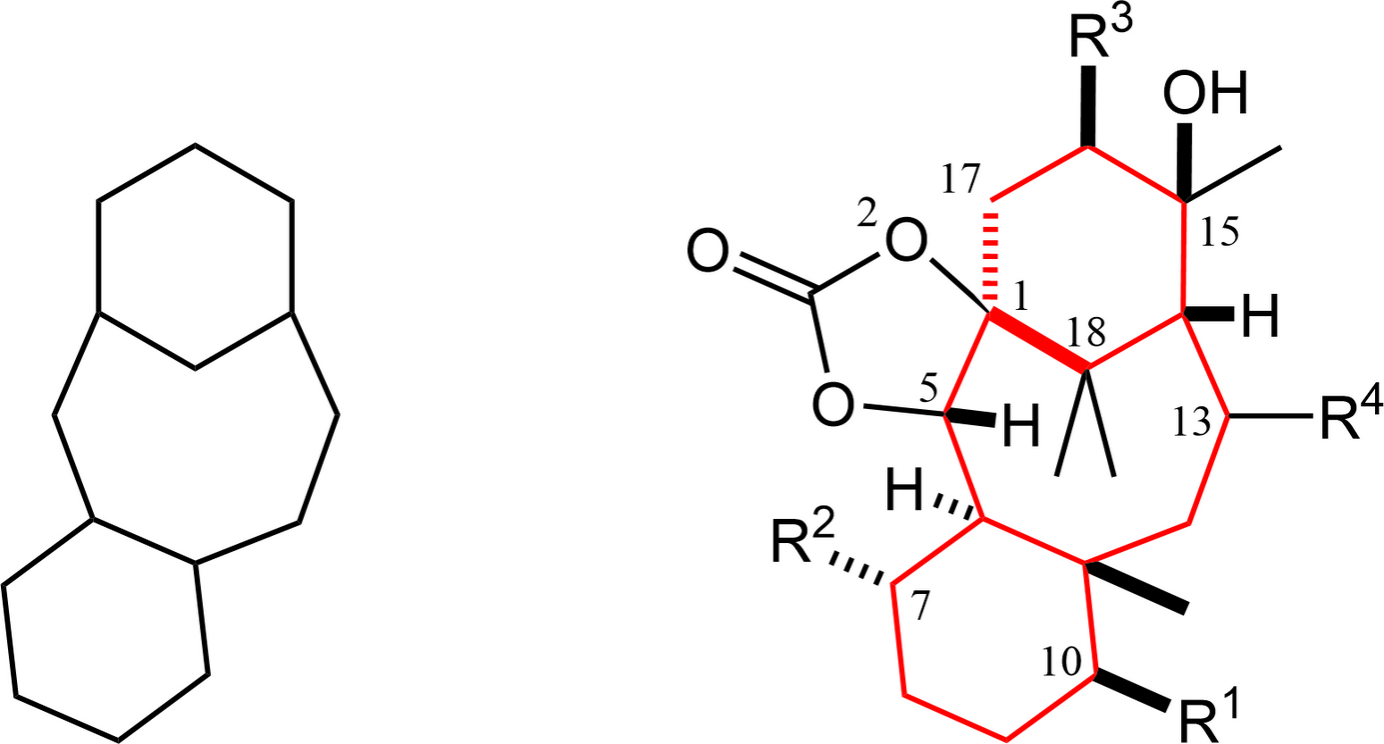
Left: Structure of the tri­cyclo­[9.3.1.0^3,8^]penta­decane (taxane) skeleton. Right: Core framework of the title compounds, indicating the taxane skeleton with red lines. *R*^1^ = OC(=O)Ph, *R*^2^ = OCH_2_OCH_3_, *R*^3^ = OCH_2_Ph, *R*^4^ = OC(=O)CH_3_.

Paclitaxel (systematic name: (1*S*,2*S*,3*R*,4*S*,7*R*,9*S*,10*S*,12*R*,15*S*)-4,12-diacet­oxy-1,9-dihy­droxy-15-{[(2*R*,3*S*)-3-benzoylamino-2-hy­droxy-3-phen­yl]propano­yl}­oxy-10,14,17,17-tetra­methyl-11-oxo-6-oxa-tetra­cyclo­[11.3.1.0^3,10^.0^4,7^]hepta­dec-13-en-2-yl benzoate) is a well-known natural diterpenoid containing a taxane skeleton (tri­cyclo­[9.3.1.0^3,8^]penta­decane; Fig. 1 ▸), and exerts potent anti­tumour activity (Wall & Wani, 1995 ▸). Its complicated and highly functionalized structure with remarkable bioactivity has inspired immense chemical and medicinal inter­est. The title compounds, which are C-13 epimers of one another, were afforded in a synthetic study of paclitaxel (Fukaya *et al.*, 2015*a* ▸,*b* ▸, Iiyama, *et al.*, 2022 ▸). Previously, several closely related structures (Oishi *et al.*, 2015*a* ▸,*b* ▸, 2021 ▸) have been reported (see Section 4).
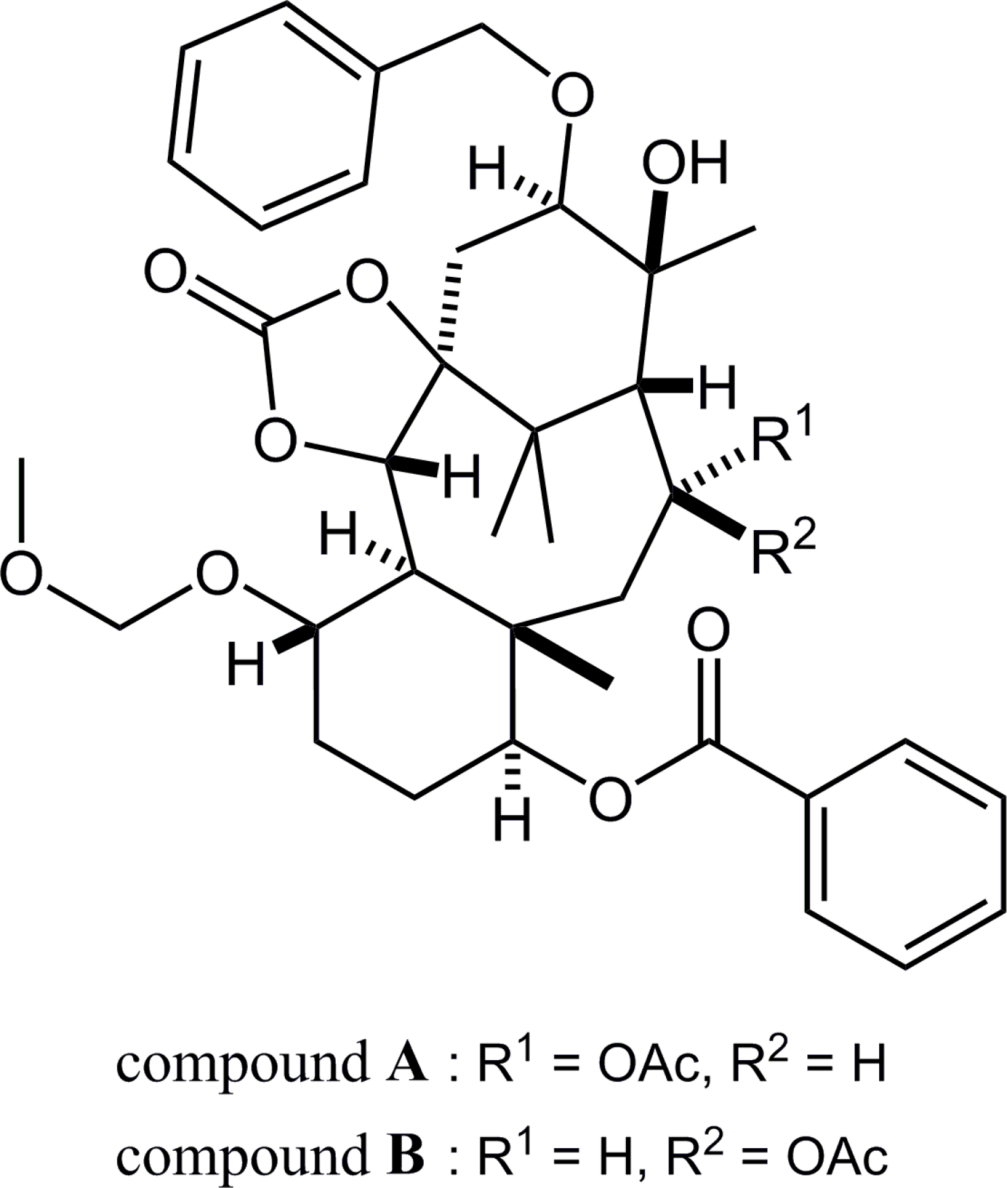


## 2. Structural commentary

**Figure 2:**
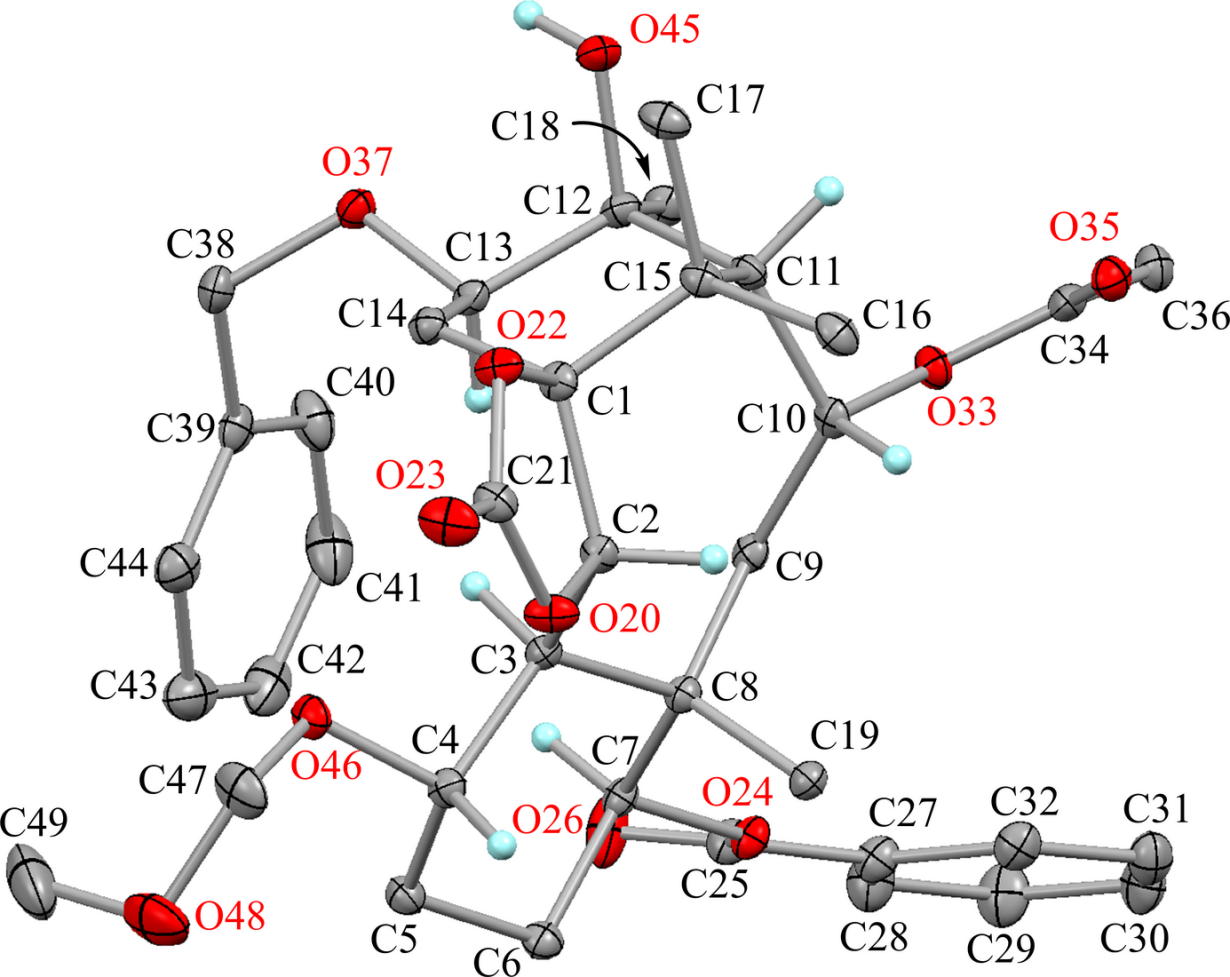
The mol­ecular structure of title compound **A** with atom labels. Displacement ellipsoids are drawn at the 30% probability level. Only the H atoms connected to O and chiral C atoms are shown for clarity.

**Figure 4:**
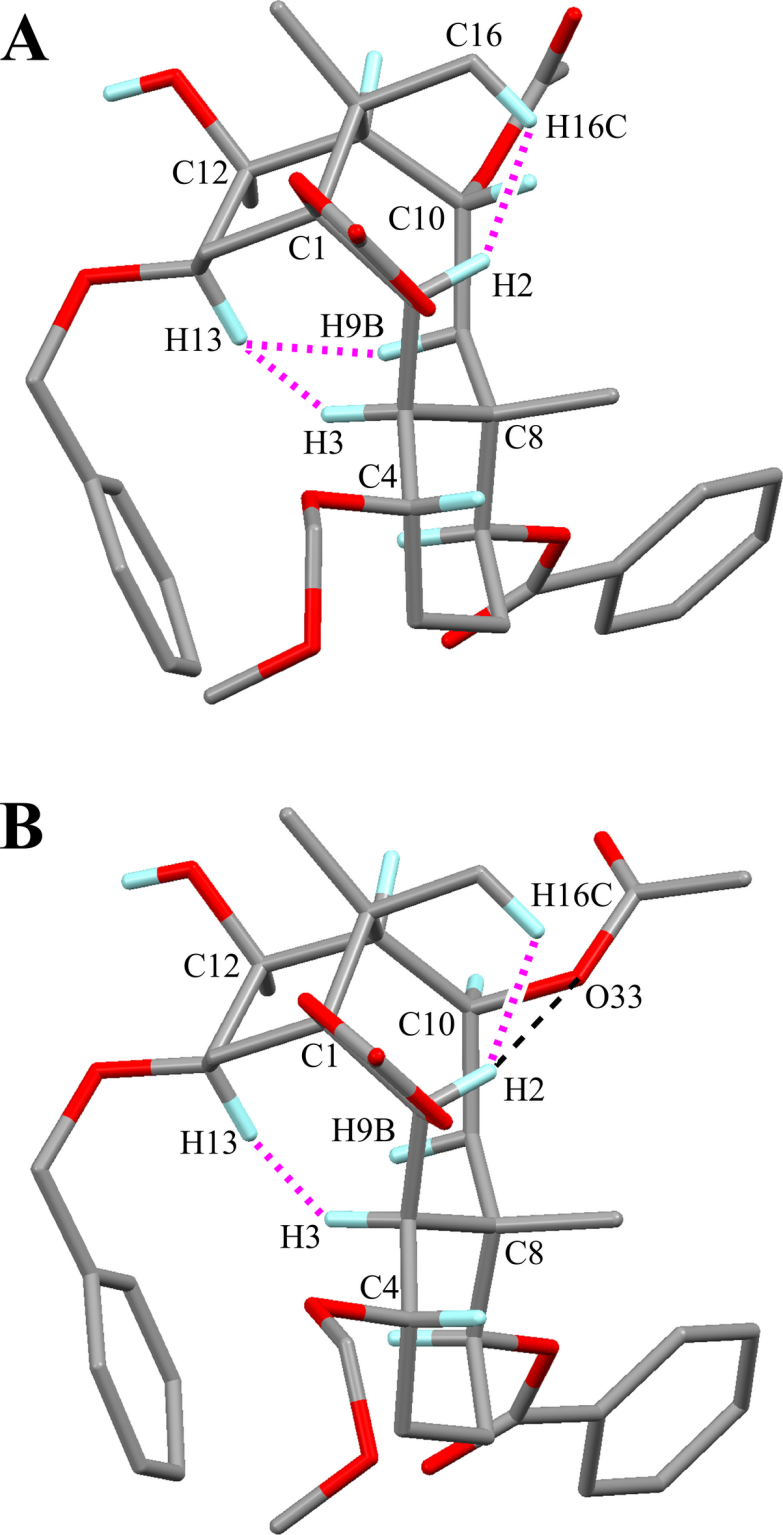
The mol­ecular conformations of the title compounds **A** and **B**, showing the intra­molecular H⋯H short contacts (purple dotted lines) and the intra­molecular C—H⋯O inter­action (black dashed line). For clarity, only H atoms involved in these inter­actions are shown.

The mol­ecular structures of the title compounds (**A**) and (**B**) are shown in Figs. 2 ▸ and 3 ▸, respectively. These conformations are similar except for the acet­oxy group (Fig. 4 ▸): three H⋯H short contacts are observed in the structure (**A**), however the intra­molecular C—H⋯O inter­action is generated by the etheric O atom of the acet­oxy group in (**B**) and one short contact has disappeared.

### 2.1. Compound **A**

The 1,3-dioxolane ring (C1/C2/O20/C21/O22) is essentially planar with a maximum deviation of −0.018 (2) Å for atom C1. The cyclo­hexane ring (C3–C8) adopts a chair form with puckering parameters *Q* = 0.559 (2) Å, *θ* = 8.6 (2)°, *φ* = 231.6 (15)°, *Q*(2) = 0.082 (2) Å and *Q*(3) = 0.553 (2) Å. The larger substituents (C3—C2, C7—O24 and C8—C9) are in equatorial positions, whereas the meth­oxy­meth­oxy group (C4—O46) is tilted from the ideal equatorial position with a dihedral angle of 57.53 (11)° to the Cremer & Pople (1975 ▸) plane. Another cyclo­hexane ring (C1/C14/C13/C12/C11/C15) also adopts a chair form with puckering parameters *Q* = 0.535 (2) Å, *θ* = 168.6 (2)°, *φ* = 108.4 (12)°, *Q*(2) = 0.104 (2) Å and *Q*(3) = −0.524 (2) Å. Owing to the *syn*-fused ring system, the sterically more hindered substituents (C1—C2 and C11—C10) and hy­droxy group (C12—O45) are in axial positions, while the benz­yloxy group (C13—O37) is slightly tilted from the ideal equatorial position with angle to the Cremer & Pople plane of 59.55 (10)°. The central cyclo­octane ring (C1–C3/C8–C11/C15) adopts a chair–chair form with puckering parameters *Q* = 0.862 (2) Å, *Q*(2) = 0.170 (2) Å, *φ*(2) = 115.3 (7)°, *Q*(3) = 0.108 (2) Å, *φ*(3) = 3.9 (11)° and *Q*(4) = 0.838 (2) Å. Unusual *sp*^3^ angles are observed at atoms C9 and C10 by strained ring system, with 125.07 (16)° for C8—C9—C10 and 124.29 (17)° for C9—C10—C11. There are intra­molecular short contacts between atoms H2⋯H16*C*, H3⋯H13 and H9*B*⋯H13 with distances of 1.81, 1.94 and 1.97 Å, respectively (Fig. 4 ▸).

### 2.2. Compound **B**

The 1,3-dioxolane ring (C1/C2/O20/C21/O22) is essentially planar with a maximum deviation of −0.014 (2) Å for atom C2. The cyclo­hexane ring (C3–C8) adopts a chair form with puckering parameters *Q* = 0.573 (2) Å, *θ* = 6.9 (2)°, *φ* = 241.3 (19)°, *Q*(2) = 0.068 (2) Å and *Q*(3) = 0.569 (2) Å. The larger substituents (C3—C2, C7—O24 and C8—C9) are in equatorial positions, while the meth­oxy­meth­oxy group (C4—O46) is tilted slightly from the ideal equatorial position with a dihedral angle to the Cremer & Pople plane of 58.95 (14)°. Another cyclo­hexane ring (C1/C14/C13/C12/C11/C15) also adopts a chair form with puckering parameters *Q* = 0.556 (2)Å, *θ* = 167.1 (2)°, *φ* = 110.0 (10)°, *Q*(2) = 0.124 (2) Å and *Q*(3) = −0.542 (2) Å. Similar to compound **A**, the sterically more hindered substituents (C1—C2 and C11—C10) and hy­droxy group (C12—O45) are in axial positions because of the *syn*-fused ring system, while the benz­yloxy group (C13—O37) is in an equatorial position. The central cyclo­octane ring (C1–C3/C8–C11/C15) adopts a chair–chair form with puckering parameters *Q* = 0.825 (2) Å, *Q*(2) = 0.161 (2) Å, *φ*(2) = 113.9 (8)°, *Q*(3) = 0.166 (2) Å, *φ*(3) = 17.2 (8)° and *Q*(4) = 0.792 (2) Å. Atypical *sp*^3^ angles are observed at atoms C9 and C10 of the ring system, which is strained more than in compound **A**, with 128.84 (17)° for C8—C9—C10 and 127.33 (19)° for C9—C10—C11. The mol­ecular conformation is supported by an intra­molecular C—H⋯O hydrogen bond (C2—H2⋯O33), generating an *S*(7) graph-set motif. There are two intra­molecular short contacts between the atoms H2⋯H16*C* and H3⋯H13, with distances of 1.80 and 1.92 Å, respectively. The H9*B*⋯H13 short contact is not observed, the distance being 2.03 Å.

The benzoyl group (C25/O26/C27–C32) is disordered over two orientations, with refined occupancies of 0.499 (3) and 0.501 (3). The meth­oxy­methyl group (C47/O48/C49) is also disordered over two sites, with refined occupancies of 0.495 (4) and 0.505 (4).

## 3. Supra­molecular features

### 3.1. Compound **A**

**Figure 5:**
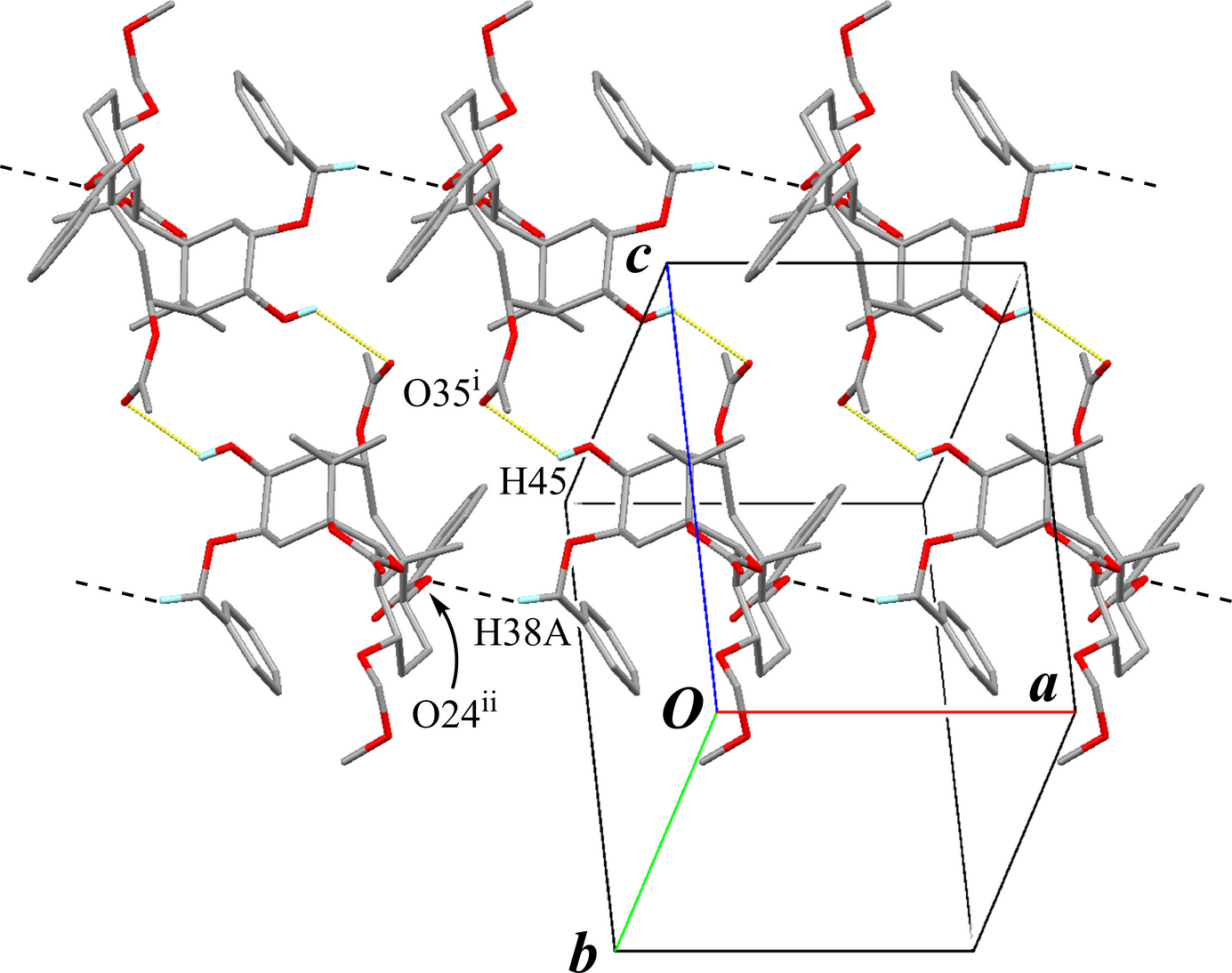
A partial packing diagram of compound **A** showing the tape structure running along the *a-*axis direction. Yellow dotted lines and black dashed lines indicate the inter­molecular O—H⋯O hydrogen bonds and C—H⋯O inter­actions, respectively. Only H atoms involved in these inter­actions are shown for clarity. [Symmetry codes: (i) −*x*, −*y* + 1, −*z* + 2; (ii) *x* − 1, *y*, *z*.]

**Figure 6:**
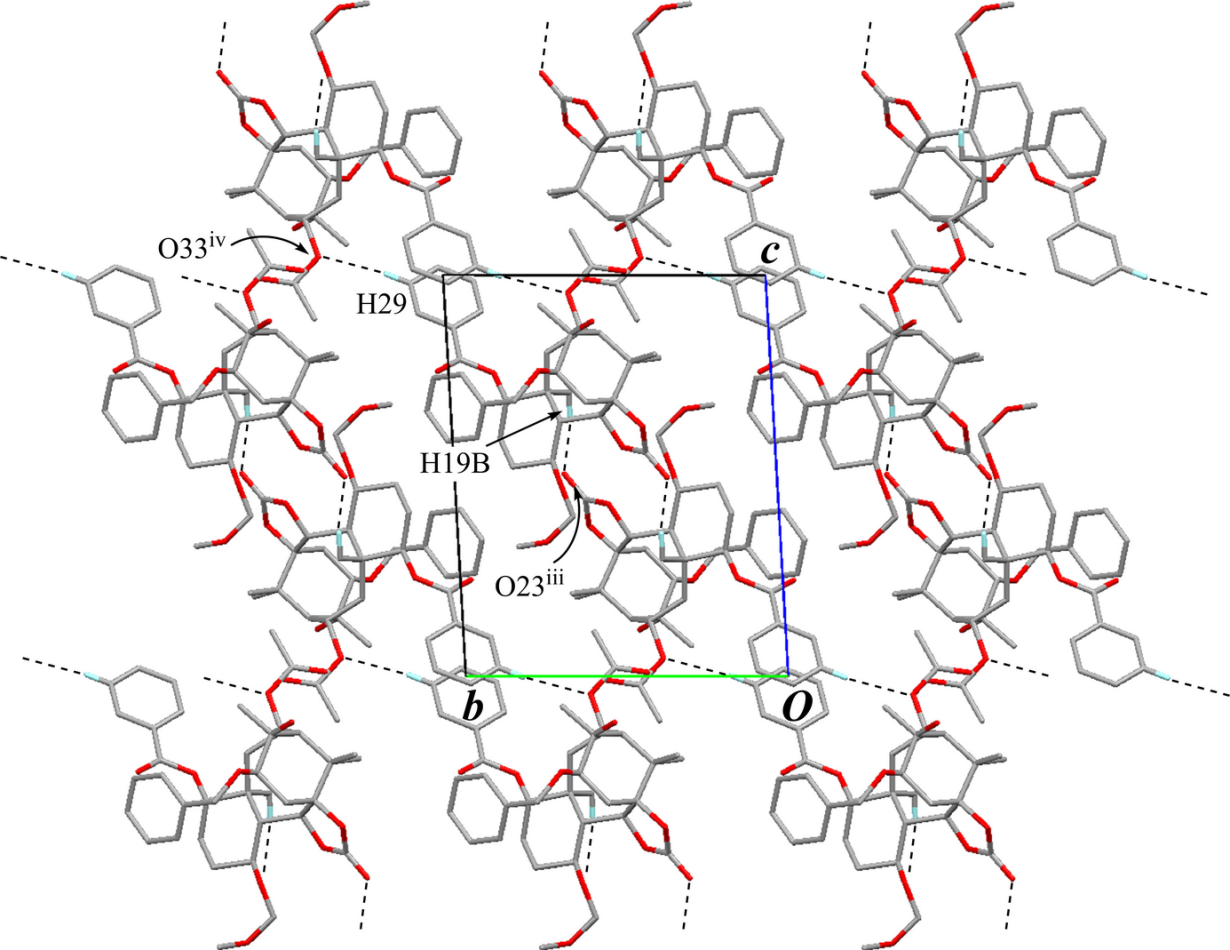
The packing of compound **A** viewed down the *a* axis. Black dashed lines indicate the inter­molecular C—H⋯O inter­actions. Only H atoms involved in hydrogen bonds are shown for clarity. [Symmetry codes: (iii) −*x* + 1, −*y* + 1, −*z* + 1; (iv) −*x* + 1, −*y* + 2, −*z* + 2.]

In the crystal, pairs of inter­molecular O—H⋯O hydrogen bonds (O45—H45⋯O35^i^; symmetry code as given in Table 1 ▸), generating an 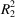
(16) graph-set motif, form inversion dimers (Fig. 5 ▸). The dimers are linked by weak inter­molecular C—H⋯O inter­actions (C38—H38*A*⋯O24^ii^; Table 1 ▸) extending a tape structure running along the *a-*axis direction. The tapes are further connected by weak inter­molecular C—H⋯O inter­actions (C19—H19*B*⋯O23^iii^ and C29—H29⋯O33^iv^; Table 1 ▸, Fig. 6 ▸) into a three-dimensional network. In addition, an inter­molecular π–π inter­action (*Cg*1⋯*Cg*1^iv^; depicted as overlapped rings at the corners of the unit cell in Fig. 6 ▸, where *Cg*1 is the centroid of the C27–C32 benzene ring) is also observed with a centroid–centroid distance of 3.7051 (15) Å.

### 3.2. Compound **B**

**Figure 7:**
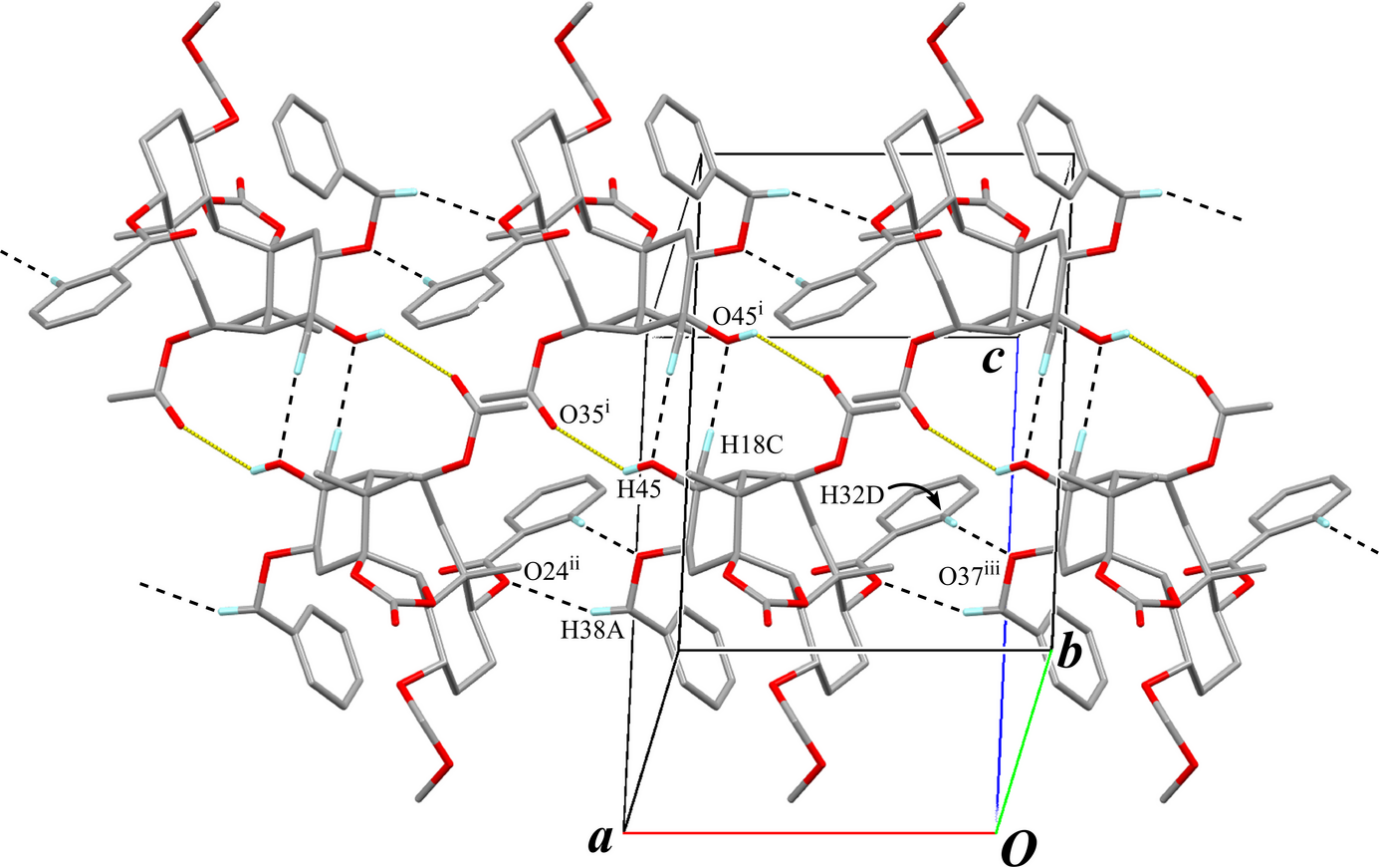
A partial packing diagram of compound **B** showing the tape structure running along the *a-*axis direction. Yellow dotted lines and black dashed lines indicate the inter­molecular O—H⋯O hydrogen bonds and C—H⋯O inter­actions, respectively. Only H atoms involved in these inter­actions are shown for clarity. [Symmetry codes: (i) −*x* + 2, −*y* + 2, −*z* + 1; (ii) *x* + 1, *y*, *z*; (iii) *x* − 1, *y*, *z*.]

**Figure 8:**
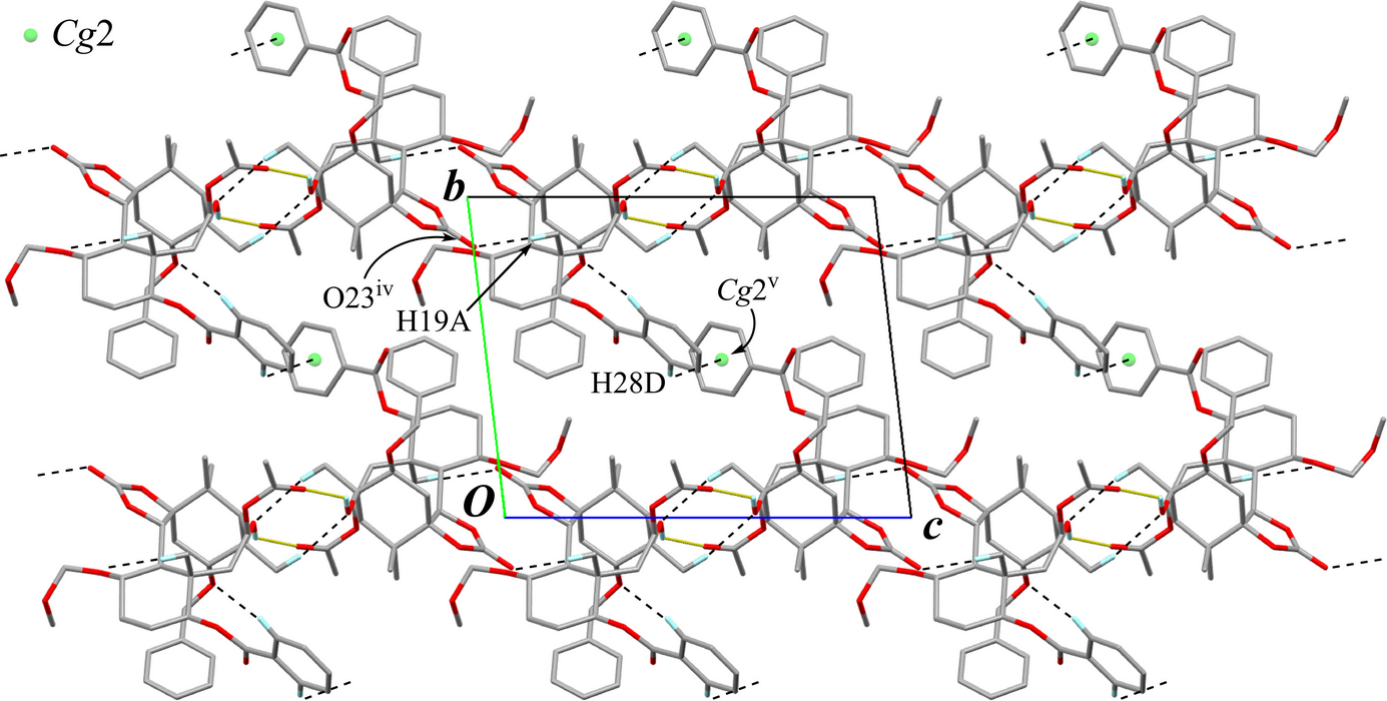
The packing of compound **B** viewed down the *a* axis. The tape structure is on the *c* axis line of the unit cell, depicted as overlapped mol­ecules including a pair of O—H⋯O hydrogen bonds (yellow dotted lines). Black dashed lines indicate the inter­molecular C—H⋯O and C—H⋯π inter­actions. Only H atoms involved in hydrogen bonds are shown for clarity. [Symmetry codes: (iv) −*x* + 1, −*y* + 2, −*z*; (v) −*x* + 1, −*y* + 1, −*z* + 1.]

The crystal packing also features pairs of inter­molecular O—H⋯O hydrogen bonds (O45—H45⋯O35^i^; Table 2 ▸) with an 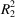
(16) graph-set motif, forming inversion dimers (Fig. 7 ▸). Pairs of inter­molecular C—H⋯O inter­actions (C18—H18*C*⋯O45^i^; Table 2 ▸) support the dimer formation, with an 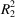
(8) graph-set motif. The dimers are linked by inter­molecular C—H⋯O inter­actions (C38—H38*A*⋯O24^ii^ and C32*D*—H32*D*⋯O37^iii^; Table 2 ▸), elongating a tape structure running along the *a-*axis direction. Adjacent tapes are further connected through weak inter­molecular C—H⋯O and C—H⋯π inter­actions (C19—H19*A*⋯O23^iv^ and C28*D*—H28*D*⋯*Cg*2^v^; Table 2 ▸, Fig. 8 ▸) into a three-dimensional architecture.

## 4. Database survey

**Figure 9:**
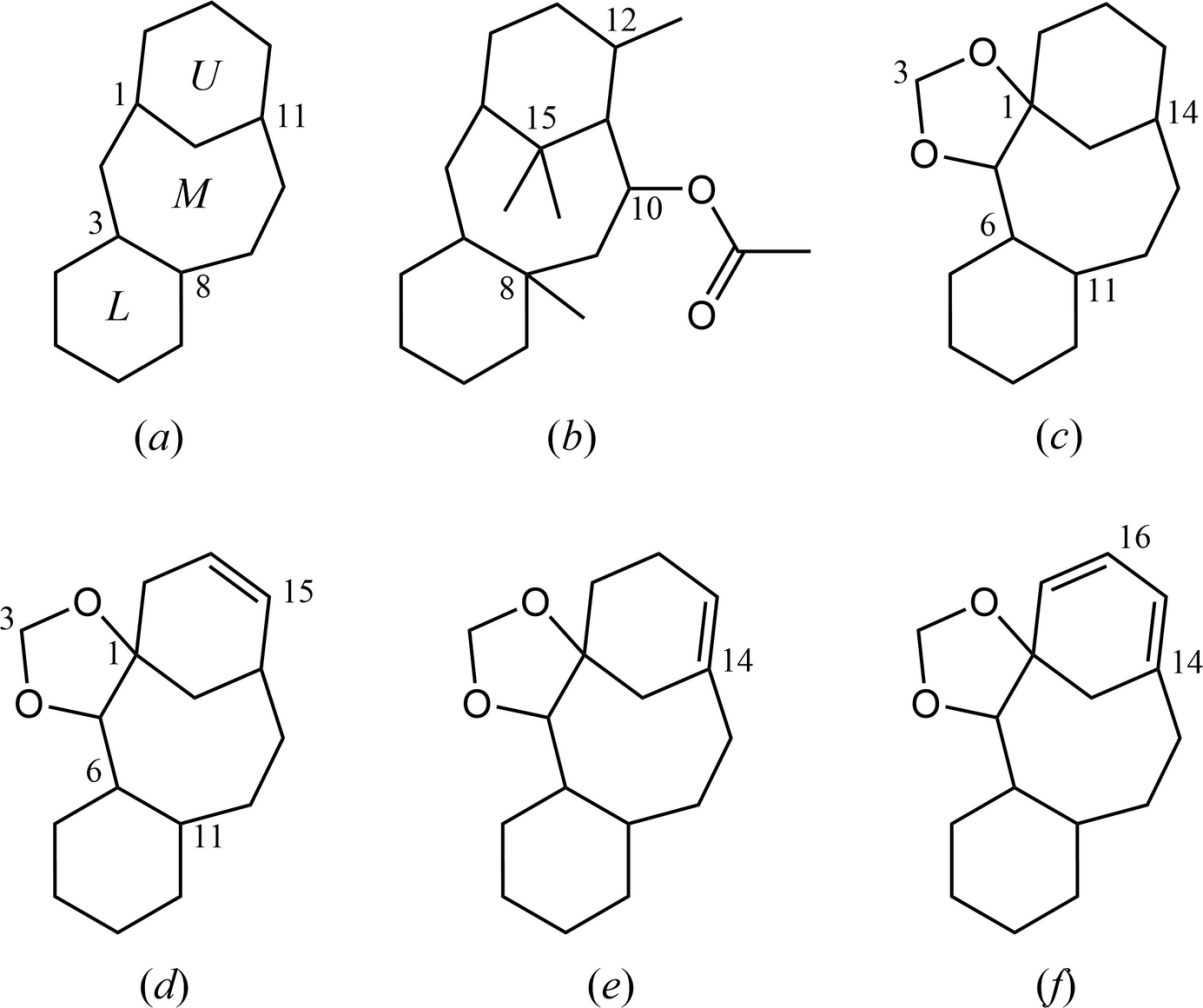
Core structures for the database survey; (*a*) tri­cyclo­[9.3.1.0^3,8^]penta­decane (taxane) with ring labelling (*U*: an upper cyclo­hexane, *M*: a middle cyclo­octane and *L*: a lower cyclo­hexa­ne) and its (*b*) 10-acet­oxy-8,12,15,15-tetra­methyl derivative, (*c*) tetra­cyclo­[12.3.1.0^1,5^.0^6,11^]octa­decane as a the main framework of the title compound, and its (*d*) 15-ene, (*e*) 14-ene and (*e*) 14,16-diene derivatives. Ring-fusion geometries in the related structures are similar to the title compound, as *syn*-*UM* and *anti*-*ML*.

In the Cambridge Structural Database (CSD, Version 5.45, September 2024; Groom *et al.*, 2016 ▸), 106 structures containing a tri­cyclo­[9.3.1.0^3,8^]penta­decane (taxane) core, (*a*), are deposited (Fig. 9 ▸). These include two chiral compounds [CSD refcodes OACBRT10 (Shiro & Koyama, 1971 ▸) and ZOPNUN (Kelly *et al.*, 1996 ▸)], possessing a 10-acet­oxy-8,12,15,15-tetra­methyl­taxane skeleton, (*b*). The ring conformations of the taxane framework [upper, cyclo­hexane (*U*), middle, cyclo­octane (*M*) and lower, cyclo­hexane, (*L*)] in the former structure are slightly skewed boat, boat–chair and chair forms, respectively, while those in the latter are chair, twist–boat–chair and half-chair, respectively. The relative stereochemistries at the C-13 acet­oxy groups in both compounds are coincident with that of compound **B**.

Another search for a saturated tetra­cyclic core related to the title compound, (*c*), gave no entries, whereas its 15-ene (*d*), 14-ene (*e*) and 14,16-diene (*f*) derivatives, afforded in our synthetic studies, are available [XULNAV, XULMOI and XULMUO (Oishi *et al.*, 2015*a* ▸), PAJKEU (Oishi *et al.*, 2021 ▸) and GUHMUD (Oishi *et al.*, 2015*b* ▸)].

## 5. Synthesis and crystallization

The title compounds were obtained in the synthetic study of paclitaxel (Fukaya *et al.*, 2015*a* ▸,*b* ▸). The precursor of the cyclo­hexane unit (C1/C14/C13/C12/C11/C15), prepared according to the reported procedure (Nicolaou *et al.*, 1995 ▸), were coupled with the substituted cyclo­hexane unit (C3–C8) derived from 3-methyl­anisole by a Shapiro reaction (Nicolaou *et al.*, 1995 ▸). A cyclization reaction generated the central cyclo­octane, and further manipulations of the functional groups gave a mixture of tetra­cyclic benzoates **A** and **B**. Separation and purification were carried out by silica gel chromatography. Colourless crystals of **A** suitable for X-ray diffraction were grown from a benzene solution under a pentane-saturated atmosphere by slow evaporation at ambient temperature. In a similar manner, colourless crystals of **B** were also obtained.

## 6. Refinement

Crystal data, data collection and structure refinement details are summarized in Table 3 ▸. C-bound H atoms were positioned geometrically with C—H = 0.95–1.00 Å, and constrained to ride on their parent atoms with *U*_iso_(H) = 1.2*U*_eq_(C) or 1.5*U*_eq_(methyl C). The H atom of the hy­droxy group was located in a difference map, and treated as riding with O—H = 0.84 Å and *U*_iso_(H) = 1.5*U*_eq_(O). One problematic reflection for **A** and four reflections for **B** were omitted in the final cycles of refinement.

## Supplementary Material

Crystal structure: contains datablock(s) global, A, B. DOI: 10.1107/S2056989024012234/ox2011sup1.cif

Structure factors: contains datablock(s) A. DOI: 10.1107/S2056989024012234/ox2011Asup2.hkl

Structure factors: contains datablock(s) B. DOI: 10.1107/S2056989024012234/ox2011Bsup3.hkl

CCDC references: 2411155, 2411154

Additional supporting information:  crystallographic information; 3D view; checkCIF report

## Figures and Tables

**Figure 3:**
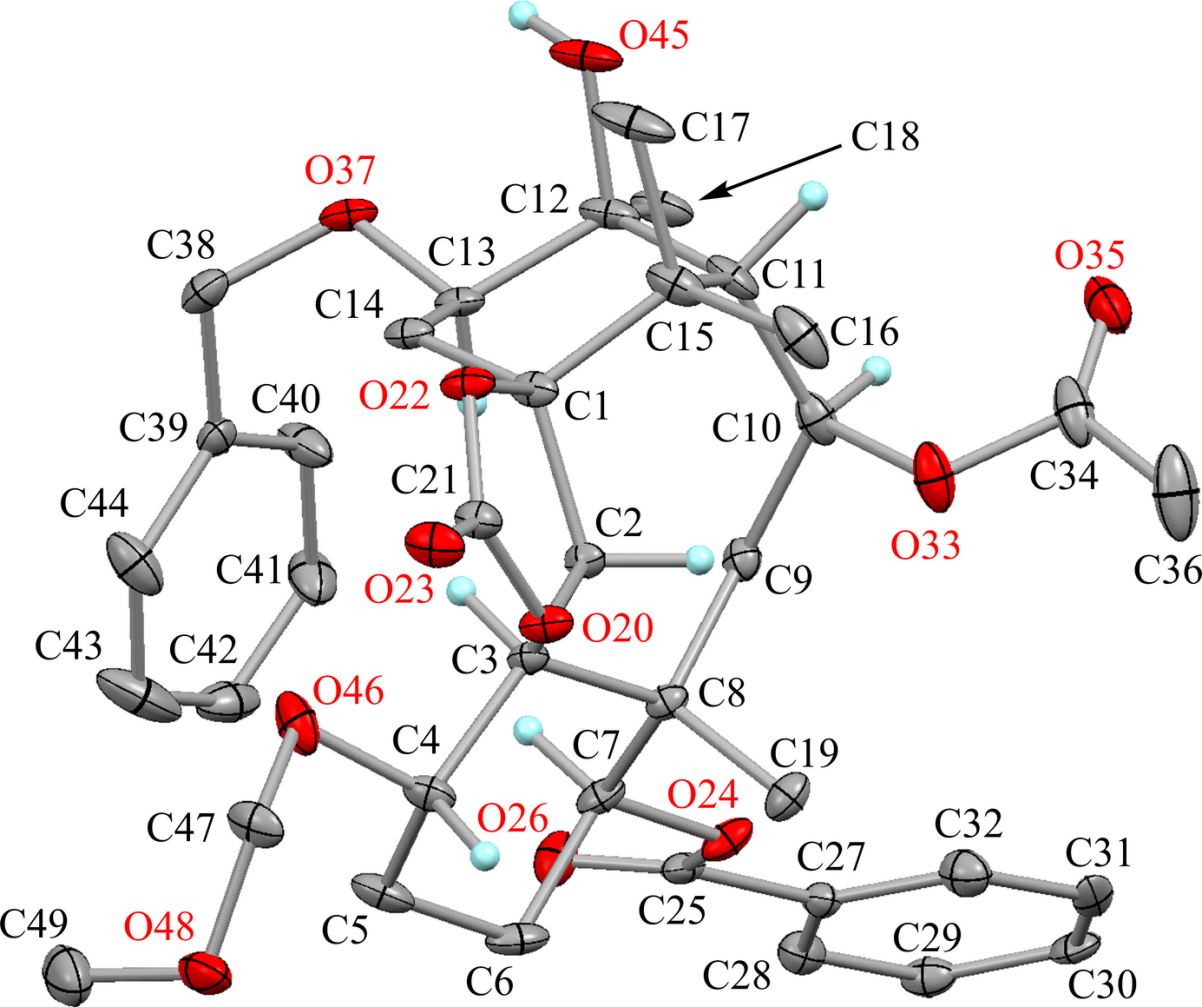
The mol­ecular structure of title compound **B** with atom labels. Displacement ellipsoids are drawn at the 30% probability level. H atoms involved in these interactions or connected to O and chiral C atoms are shown. Other possible positions of disordered atoms have been omitted.

**Table 1:** Hydrogen-bond geometry (Å, °) for **A**[Chem scheme1]

*D*—H⋯*A*	*D*—H	H⋯*A*	*D*⋯*A*	*D*—H⋯*A*
O45—H45⋯O35^i^	0.84	2.50	3.048 (2)	124
C38—H38*A*⋯O24^ii^	0.99	2.54	3.483 (3)	159
C19—H19*A*⋯O23^iii^	0.98	2.55	3.496 (3)	163
C29—H29⋯O33^iv^	0.95	2.57	3.510 (3)	173

Symmetry codes: (i) 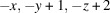; (ii) 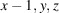; (iii) 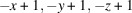; (iv) 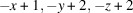.

**Table 2:** Hydrogen-bond geometry (Å, °) for **B**[Chem scheme1] *Cg*2 is the centroid of the C27–C32 benzene ring.

*D*—H⋯*A*	*D*—H	H⋯*A*	*D*⋯*A*	*D*—H⋯*A*
C2—H2⋯O33	1.00	2.44	3.344 (3)	150
O45—H45⋯O35^i^	0.84	2.43	3.098 (2)	137
C18—H18*C*⋯O45^i^	0.98	2.47	3.442 (3)	169
C38—H38*A*⋯O24^ii^	0.99	2.48	3.409 (3)	156
C32*D*—H32*D*⋯O37^iii^	0.95	2.57	3.465 (16)	157
C19—H19*A*⋯O23^iv^	0.98	2.60	3.560 (3)	168
C28*D*—H28*D*⋯*Cg*2^v^	0.95	2.93	3.546 (7)	124

Symmetry codes: (i) 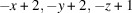; (ii) 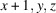; (iii) 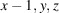; (iv) 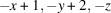; (v) 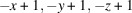.

**Table 3:** Experimental details

	**A**	**B**
Crystal data		
Chemical formula	C_38_H_48_O_11_	C_38_H_48_O_11_
*M*_r_	680.76	680.76
Crystal system, space group	Triclinic, *P* 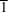	Triclinic, *P* 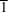
Temperature (K)	90	90
*a*, *b*, *c* (Å)	9.8868 (5), 11.7682 (5), 14.6899 (7)	9.6913 (7), 11.8313 (8), 14.9295 (9)
α, β, γ (°)	86.598 (2), 85.322 (1), 89.188 (1)	96.304 (2), 94.004 (2), 93.651 (2)
*V* (Å^3^)	1700.40 (14)	1692.9 (2)
*Z*	2	2
Radiation type	Mo *K*α	Mo *K*α
μ (mm^−1^)	0.10	0.10
Crystal size (mm)	0.34 × 0.30 × 0.17	0.19 × 0.17 × 0.09

Data collection		
Diffractometer	Bruker D8 Venture	Bruker D8 Venture
Absorption correction	Multi-scan (*SADABS*; Krause *et al.*, 2015 ▸)	Multi-scan (*SADABS*; Krause *et al.*, 2015 ▸)
*T*_min_, *T*_max_	0.97, 0.98	0.98, 0.99
No. of measured, independent and observed [*I* > 2σ(*I*)] reflections	30870, 5924, 4770	15750, 5809, 3577
*R*_int_	0.033	0.090
(sin θ/λ)_max_ (Å^−1^)	0.595	0.595

Refinement		
*R*[*F*^2^ > 2σ(*F*^2^)], *wR*(*F*^2^), *S*	0.046, 0.095, 1.05	0.045, 0.096, 1.03
No. of reflections	5924	5809
No. of parameters	449	551
No. of restraints	8	81
H-atom treatment	H-atom parameters constrained	H-atom parameters constrained
Δρ_max_, Δρ_min_ (e Å^−3^)	0.59, −0.27	0.26, −0.22

Computer programs: *APEX4* and *SAINT* (Bruker, 2021 ▸), *SHELXT2019* (Sheldrick, 2015*a* ▸), *SHELXL2019* (Sheldrick, 2015*b* ▸), *Mercury* (Macrae *et al.*, 2020 ▸), *publCIF* (Westrip, 2010 ▸) and *PLATON* (Spek, 2020 ▸).

## supplementary crystallographic information

### (±)-(1SR,5SR,6SR,7SR,10SR,11SR,13RS,14SR,15SR,16RS)-13-Acetoxy-16-benzyloxy-15-hydroxy-7-methoxymethoxy-3-oxo-11,15,18,18-tetramethyl-2,4-dioxatetracyclo[12.3.1.01,5.06,11]octadecan-10-yl benzoate (A) Crystal data

**Table:**

C_38_H_48_O_11_	*F*(000) = 728
*M**_r_* = 680.76	*D*_x_ = 1.330 Mg m^−^^3^
Triclinic, *P*1	Melting point = 488.5–490.5 K
*a* = 9.8868 (5) Å	Mo *K*α radiation, λ = 0.71073 Å
*b* = 11.7682 (5) Å	Cell parameters from 9886 reflections
*c* = 14.6899 (7) Å	θ = 2.4–25.1°
α = 86.598 (2)°	µ = 0.10 mm^−^^1^
β = 85.322 (1)°	*T* = 90 K
γ = 89.188 (1)°	Prism, colourless
*V* = 1700.40 (14) Å^3^	0.34 × 0.30 × 0.17 mm
*Z* = 2	

### (±)-(1SR,5SR,6SR,7SR,10SR,11SR,13RS,14SR,15SR,16RS)-13-Acetoxy-16-benzyloxy-15-hydroxy-7-methoxymethoxy-3-oxo-11,15,18,18-tetramethyl-2,4-dioxatetracyclo[12.3.1.01,5.06,11]octadecan-10-yl benzoate (A) Data collection

**Table:**

Bruker D8 Venture diffractometer	5924 independent reflections
Radiation source: fine-focus sealed tube	4770 reflections with *I* > 2σ(*I*)
Multilayered confocal mirror monochromator	*R*_int_ = 0.033
Detector resolution: 10.4167 pixels mm^-1^	θ_max_ = 25.0°, θ_min_ = 2.4°
φ and ω scans	*h* = −11→11
Absorption correction: multi-scan (SADABS; Krause *et al.*, 2015)	*k* = −14→14
*T*_min_ = 0.97, *T*_max_ = 0.98	*l* = −17→17
30870 measured reflections	

### (±)-(1SR,5SR,6SR,7SR,10SR,11SR,13RS,14SR,15SR,16RS)-13-Acetoxy-16-benzyloxy-15-hydroxy-7-methoxymethoxy-3-oxo-11,15,18,18-tetramethyl-2,4-dioxatetracyclo[12.3.1.01,5.06,11]octadecan-10-yl benzoate (A) Refinement

**Table:**

Refinement on *F*^2^	Primary atom site location: structure-invariant direct methods
Least-squares matrix: full	Secondary atom site location: difference Fourier map
*R*[*F*^2^ > 2σ(*F*^2^)] = 0.046	Hydrogen site location: inferred from neighbouring sites
*wR*(*F*^2^) = 0.095	H-atom parameters constrained
*S* = 1.05	*w* = 1/[σ^2^(*F*_o_^2^) + 2.3674*P*] where *P* = (*F*_o_^2^ + 2*F*_c_^2^)/3
5924 reflections	(Δ/σ)_max_ < 0.001
449 parameters	Δρ_max_ = 0.59 e Å^−^^3^
8 restraints	Δρ_min_ = −0.27 e Å^−^^3^

### (±)-(1SR,5SR,6SR,7SR,10SR,11SR,13RS,14SR,15SR,16RS)-13-Acetoxy-16-benzyloxy-15-hydroxy-7-methoxymethoxy-3-oxo-11,15,18,18-tetramethyl-2,4-dioxatetracyclo[12.3.1.01,5.06,11]octadecan-10-yl benzoate (A) Special details

**Table:**

Experimental. IR (film): 3527, 2947, 1799, 1720, 1270, 1099, 1041 cm^-1^; ^1^H NMR (500 MHz, CDCl_3_): *δ* (p.p.m.) 7.98–7.94 (m, 2H), 7.61–7.42 (m, 8H), 5.47 (d, *J* = 9.7 Hz, 1H), 4.84 (d, *J* = 12.6 Hz, 1H), 4.79 (d, *J* = 7.2 Hz, 1H), 4.70 (d, *J* = 5.4 Hz, 1H), 4.68 (d, *J* = 12.6 Hz, 1H), 4.60 (d, *J* = 7.2 Hz, 1H), 4.18 (dd, *J* = 11.6, 4.3 Hz, 1H), 3.81 (dd, *J* = 11.3, 6.6 Hz, 1H), 3.49 (ddd, *J* = 10.5, 10.5, 5.4 Hz, 1H), 3.40 (s, 3H), 2.72 (s, 1H), 2.39 (dd, *J* = 13.8, 6.6 Hz, 1H), 2.32–2.25 (m, 1H), 2.26 (dd, *J* = 13.8, 11.3 Hz, 1H), 1.98 (s, 1H), 1.87 (s, 3H), 1.83 (dddd, *J* = 13.0, 4.3, 3.7, 3.4 Hz, 1H), 1.69–1.54 (m, 4H), 1.49 (s, 3H), 1.36 (s, 3H), 1.36–1.29 (m, 1H), 1.29 (s, 3H), 1.25 (s, 3H); HRMS (ESI): *m/z* calcd for C_38_H_48_O_11_Na^+^ [*M* + Na]^+^ 703.3094, found 703.3098.
Geometry. All esds (except the esd in the dihedral angle between two l.s. planes) are estimated using the full covariance matrix. The cell esds are taken into account individually in the estimation of esds in distances, angles and torsion angles; correlations between esds in cell parameters are only used when they are defined by crystal symmetry. An approximate (isotropic) treatment of cell esds is used for estimating esds involving l.s. planes.
Refinement. Refinement of *F*^2^ against ALL reflections. The weighted *R*-factor *wR* and goodness of fit *S* are based on *F*^2^, conventional *R*-factors *R* are based on *F*, with *F* set to zero for negative *F*^2^. The threshold expression of *F*^2^ > 2*σ*(*F*^2^) is used only for calculating *R*-factors(gt) *etc.* and is not relevant to the choice of reflections for refinement. *R*-factors based on *F*^2^ are statistically about twice as large as those based on *F*, and *R*-factors based on ALL data will be even larger. Model structure was improved by utilizing the BUMP command to solve the intramolecular short contact of 1.78 Å between atoms H2 and H16C. Problematic reflection with |I(obs)–I(calc)|/σW(I) greater than 10 (0 –1 1) have been omitted in the final refinement.

### (±)-(1SR,5SR,6SR,7SR,10SR,11SR,13RS,14SR,15SR,16RS)-13-Acetoxy-16-benzyloxy-15-hydroxy-7-methoxymethoxy-3-oxo-11,15,18,18-tetramethyl-2,4-dioxatetracyclo[12.3.1.01,5.06,11]octadecan-10-yl benzoate (A) Fractional atomic coordinates and isotropic or equivalent isotropic displacement parameters (Å^2^)

**Table:**

	*x*	*y*	*z*	*U*_iso_*/*U*_eq_	
C1	0.1575 (2)	0.49329 (17)	0.68895 (13)	0.0181 (4)	
C2	0.3012 (2)	0.53459 (17)	0.65228 (14)	0.0180 (4)	
H2	0.364627	0.504169	0.697712	0.022000*	
C3	0.3312 (2)	0.66221 (16)	0.63610 (13)	0.0163 (4)	
H3	0.242916	0.703436	0.647736	0.020000*	
C4	0.3825 (2)	0.69363 (18)	0.53662 (14)	0.0196 (5)	
H4	0.458704	0.641784	0.516406	0.024000*	
C5	0.4260 (2)	0.81720 (17)	0.52214 (14)	0.0214 (5)	
H5A	0.344254	0.866810	0.525774	0.026000*	
H5B	0.472073	0.829490	0.459994	0.026000*	
C6	0.5212 (2)	0.85177 (18)	0.59215 (14)	0.0213 (5)	
H6A	0.608040	0.809201	0.584255	0.026000*	
H6B	0.540739	0.934114	0.582965	0.026000*	
C7	0.4547 (2)	0.82591 (17)	0.68737 (14)	0.0188 (4)	
H7	0.367636	0.869937	0.694442	0.023000*	
C8	0.4250 (2)	0.69805 (17)	0.70927 (13)	0.0171 (4)	
C9	0.3523 (2)	0.69204 (17)	0.80703 (13)	0.0182 (4)	
H9A	0.413003	0.729958	0.845931	0.022000*	
H9B	0.270565	0.741282	0.803822	0.022000*	
C10	0.3049 (2)	0.58120 (17)	0.86171 (13)	0.0184 (4)	
H10	0.375931	0.521219	0.850473	0.022000*	
C11	0.1628 (2)	0.52615 (17)	0.85707 (13)	0.0183 (4)	
H11	0.146310	0.482755	0.917492	0.022000*	
C12	0.0423 (2)	0.61215 (18)	0.85503 (14)	0.0192 (4)	
C13	0.0287 (2)	0.66499 (17)	0.75861 (14)	0.0180 (4)	
H13	0.100467	0.723941	0.744609	0.022000*	
C14	0.0413 (2)	0.57966 (18)	0.68323 (14)	0.0195 (5)	
H14A	−0.044932	0.537561	0.685157	0.023000*	
H14B	0.053041	0.622825	0.623235	0.023000*	
C15	0.1581 (2)	0.43255 (17)	0.78513 (14)	0.0199 (5)	
C16	0.2763 (2)	0.34590 (18)	0.79403 (15)	0.0254 (5)	
H16A	0.270013	0.287310	0.749904	0.038000*	
H16B	0.270775	0.310131	0.856177	0.038000*	
H16C	0.363070	0.385421	0.781653	0.038000*	
C17	0.0308 (2)	0.35805 (18)	0.80290 (15)	0.0258 (5)	
H17A	−0.049757	0.403719	0.789971	0.039000*	
H17B	0.022566	0.328995	0.867001	0.039000*	
H17C	0.038722	0.294007	0.763023	0.039000*	
C18	0.0476 (2)	0.70511 (18)	0.92345 (14)	0.0229 (5)	
H18A	−0.035475	0.751544	0.922975	0.034000*	
H18B	0.126474	0.753477	0.906164	0.034000*	
H18C	0.055024	0.669719	0.984950	0.034000*	
C19	0.5592 (2)	0.63053 (17)	0.70719 (14)	0.0196 (4)	
H19A	0.600737	0.631588	0.644257	0.029000*	
H19B	0.541468	0.551677	0.729771	0.029000*	
H19C	0.620954	0.665246	0.746204	0.029000*	
O20	0.33177 (15)	0.47247 (12)	0.57060 (10)	0.0240 (3)	
C21	0.2303 (2)	0.40242 (17)	0.55847 (14)	0.0217 (5)	
O22	0.13024 (14)	0.40834 (12)	0.62397 (9)	0.0221 (3)	
O23	0.23124 (17)	0.34126 (13)	0.49619 (10)	0.0319 (4)	
O24	0.54321 (14)	0.85971 (12)	0.75536 (10)	0.0203 (3)	
C25	0.5220 (2)	0.96282 (18)	0.78946 (15)	0.0245 (5)	
O26	0.44533 (18)	1.03321 (13)	0.76022 (12)	0.0391 (4)	
C27	0.6043 (2)	0.97733 (19)	0.86852 (15)	0.0250 (5)	
C28	0.6035 (3)	1.0843 (2)	0.90414 (17)	0.0334 (6)	
H28	0.553417	1.145061	0.877119	0.040000*	
C29	0.6753 (3)	1.1024 (2)	0.97857 (17)	0.0376 (6)	
H29	0.675890	1.175875	1.002087	0.045000*	
C30	0.7462 (3)	1.0138 (2)	1.01883 (17)	0.0358 (6)	
H30	0.796019	1.026332	1.069877	0.043000*	
C31	0.7448 (2)	0.9068 (2)	0.98505 (16)	0.0310 (5)	
H31	0.792449	0.845681	1.013621	0.037000*	
C32	0.6744 (2)	0.88823 (19)	0.90993 (15)	0.0259 (5)	
H32	0.674031	0.814604	0.886705	0.031000*	
O33	0.31100 (15)	0.61637 (12)	0.95551 (9)	0.0218 (3)	
C34	0.3376 (2)	0.53730 (19)	1.02166 (15)	0.0225 (5)	
O35	0.35454 (15)	0.43694 (12)	1.01003 (10)	0.0238 (3)	
C36	0.3458 (2)	0.59324 (19)	1.11001 (15)	0.0260 (5)	
H36A	0.337673	0.535469	1.160973	0.039000*	
H36B	0.271820	0.649096	1.117528	0.039000*	
H36C	0.433100	0.631760	1.109339	0.039000*	
O37	−0.10091 (14)	0.72032 (12)	0.76170 (10)	0.0219 (3)	
C38	−0.1189 (2)	0.80087 (18)	0.68634 (15)	0.0232 (5)	
H38A	−0.211348	0.834205	0.693918	0.028000*	
H38B	−0.111800	0.760273	0.628987	0.028000*	
C39	−0.0170 (2)	0.89580 (18)	0.67760 (15)	0.0232 (5)	
C40	0.0046 (3)	0.9591 (2)	0.75219 (17)	0.0348 (6)	
H40	−0.044421	0.941672	0.809543	0.042000*	
C41	0.0967 (3)	1.0467 (2)	0.7434 (2)	0.0443 (7)	
H41	0.112008	1.088431	0.794945	0.053000*	
C42	0.1666 (3)	1.0742 (2)	0.6600 (2)	0.0419 (7)	
H42	0.229621	1.134916	0.654083	0.050000*	
C43	0.1449 (3)	1.0135 (2)	0.58589 (19)	0.0380 (6)	
H43	0.191258	1.033425	0.528048	0.046000*	
C44	0.0552 (2)	0.92290 (19)	0.59516 (16)	0.0295 (5)	
H44	0.043462	0.879205	0.544074	0.035000*	
O45	−0.07714 (14)	0.54743 (12)	0.88428 (10)	0.0236 (3)	
H45	−0.145818	0.590198	0.883259	0.035000*	
O46	0.26586 (15)	0.67567 (12)	0.48682 (9)	0.0235 (3)	
C47	0.2924 (3)	0.6388 (2)	0.39841 (15)	0.0366 (6)	
H47A	0.208804	0.605322	0.379040	0.044000*	
H47B	0.362640	0.578040	0.399491	0.044000*	
O48	0.3365 (2)	0.72544 (19)	0.33378 (12)	0.0532 (6)	
C49	0.2364 (4)	0.8123 (3)	0.3229 (2)	0.0648 (10)	
H49A	0.148650	0.777361	0.316061	0.097000*	
H49B	0.262625	0.861366	0.268212	0.097000*	
H49C	0.228982	0.857890	0.376760	0.097000*	

### (±)-(1SR,5SR,6SR,7SR,10SR,11SR,13RS,14SR,15SR,16RS)-13-Acetoxy-16-benzyloxy-15-hydroxy-7-methoxymethoxy-3-oxo-11,15,18,18-tetramethyl-2,4-dioxatetracyclo[12.3.1.01,5.06,11]octadecan-10-yl benzoate (A) Atomic displacement parameters (Å^2^)

**Table:**

	*U*^11^	*U*^22^	*U*^33^	*U*^12^	*U*^13^	*U*^23^
C1	0.0206 (11)	0.0173 (11)	0.0171 (11)	−0.0027 (8)	−0.0023 (9)	−0.0052 (8)
C2	0.0196 (11)	0.0183 (11)	0.0162 (10)	0.0006 (8)	−0.0007 (8)	−0.0043 (8)
C3	0.0150 (10)	0.0166 (11)	0.0175 (10)	−0.0007 (8)	−0.0016 (8)	−0.0021 (8)
C4	0.0197 (11)	0.0219 (11)	0.0176 (11)	−0.0010 (9)	−0.0017 (9)	−0.0041 (9)
C5	0.0245 (12)	0.0208 (11)	0.0189 (11)	−0.0015 (9)	−0.0019 (9)	0.0002 (9)
C6	0.0221 (12)	0.0171 (11)	0.0244 (12)	−0.0021 (9)	−0.0017 (9)	0.0000 (9)
C7	0.0165 (11)	0.0186 (11)	0.0226 (11)	−0.0009 (8)	−0.0059 (9)	−0.0058 (9)
C8	0.0177 (11)	0.0159 (10)	0.0179 (11)	−0.0011 (8)	−0.0025 (8)	−0.0024 (8)
C9	0.0185 (11)	0.0191 (11)	0.0179 (11)	−0.0006 (8)	−0.0035 (8)	−0.0048 (8)
C10	0.0210 (11)	0.0220 (11)	0.0129 (10)	0.0016 (9)	−0.0024 (8)	−0.0052 (8)
C11	0.0200 (11)	0.0211 (11)	0.0136 (10)	−0.0020 (9)	0.0004 (8)	−0.0002 (8)
C12	0.0162 (11)	0.0226 (11)	0.0186 (11)	−0.0012 (9)	0.0008 (8)	−0.0040 (9)
C13	0.0148 (10)	0.0210 (11)	0.0189 (11)	0.0001 (8)	−0.0026 (8)	−0.0033 (8)
C14	0.0174 (11)	0.0236 (11)	0.0181 (11)	−0.0033 (9)	−0.0022 (8)	−0.0035 (9)
C15	0.0218 (11)	0.0174 (11)	0.0205 (11)	−0.0021 (9)	0.0001 (9)	−0.0019 (8)
C16	0.0337 (13)	0.0178 (11)	0.0243 (12)	−0.0002 (9)	−0.0003 (10)	0.0008 (9)
C17	0.0329 (13)	0.0215 (12)	0.0224 (12)	−0.0070 (10)	0.0036 (10)	−0.0031 (9)
C18	0.0224 (12)	0.0275 (12)	0.0194 (11)	0.0021 (9)	−0.0008 (9)	−0.0065 (9)
C19	0.0181 (11)	0.0200 (11)	0.0210 (11)	−0.0005 (9)	−0.0026 (9)	−0.0019 (9)
O20	0.0257 (8)	0.0198 (8)	0.0259 (8)	−0.0033 (6)	0.0076 (6)	−0.0098 (6)
C21	0.0266 (12)	0.0174 (11)	0.0211 (11)	−0.0017 (9)	−0.0011 (9)	−0.0020 (9)
O22	0.0223 (8)	0.0243 (8)	0.0206 (8)	−0.0058 (6)	0.0015 (6)	−0.0105 (6)
O23	0.0419 (10)	0.0285 (9)	0.0259 (9)	−0.0066 (7)	0.0044 (7)	−0.0144 (7)
O24	0.0200 (8)	0.0181 (8)	0.0240 (8)	−0.0008 (6)	−0.0059 (6)	−0.0063 (6)
C25	0.0258 (12)	0.0188 (11)	0.0299 (13)	−0.0013 (9)	−0.0051 (10)	−0.0049 (9)
O26	0.0479 (11)	0.0223 (9)	0.0522 (11)	0.0100 (8)	−0.0297 (9)	−0.0121 (8)
C27	0.0249 (12)	0.0234 (12)	0.0278 (12)	−0.0002 (9)	−0.0049 (10)	−0.0066 (10)
C28	0.0389 (15)	0.0263 (13)	0.0371 (14)	0.0034 (11)	−0.0124 (11)	−0.0080 (11)
C29	0.0462 (16)	0.0300 (14)	0.0397 (15)	−0.0014 (12)	−0.0139 (12)	−0.0132 (11)
C30	0.0392 (15)	0.0406 (15)	0.0304 (13)	−0.0002 (12)	−0.0141 (11)	−0.0097 (11)
C31	0.0315 (13)	0.0348 (14)	0.0273 (13)	0.0075 (11)	−0.0049 (10)	−0.0052 (10)
C32	0.0270 (13)	0.0238 (12)	0.0276 (12)	0.0036 (10)	−0.0030 (10)	−0.0080 (10)
O33	0.0259 (8)	0.0234 (8)	0.0166 (7)	−0.0009 (6)	−0.0041 (6)	−0.0017 (6)
C34	0.0145 (11)	0.0288 (13)	0.0237 (12)	−0.0008 (9)	−0.0022 (9)	0.0023 (10)
O35	0.0241 (8)	0.0203 (9)	0.0269 (8)	0.0025 (6)	−0.0030 (6)	0.0001 (6)
C36	0.0272 (13)	0.0303 (13)	0.0212 (12)	0.0000 (10)	−0.0063 (9)	−0.0003 (9)
O37	0.0168 (8)	0.0250 (8)	0.0239 (8)	0.0026 (6)	−0.0020 (6)	−0.0018 (6)
C38	0.0217 (12)	0.0247 (12)	0.0242 (12)	0.0047 (9)	−0.0074 (9)	−0.0023 (9)
C39	0.0219 (12)	0.0186 (11)	0.0300 (12)	0.0065 (9)	−0.0083 (10)	−0.0016 (9)
C40	0.0518 (16)	0.0236 (13)	0.0303 (14)	0.0021 (11)	−0.0122 (12)	0.0003 (10)
C41	0.068 (2)	0.0211 (13)	0.0483 (17)	−0.0027 (13)	−0.0309 (15)	−0.0008 (12)
C42	0.0390 (15)	0.0218 (13)	0.066 (2)	−0.0017 (11)	−0.0197 (14)	0.0074 (13)
C43	0.0308 (14)	0.0282 (14)	0.0529 (17)	0.0041 (11)	0.0017 (12)	0.0069 (12)
C44	0.0292 (13)	0.0250 (13)	0.0341 (14)	0.0049 (10)	−0.0029 (11)	−0.0021 (10)
O45	0.0177 (8)	0.0279 (8)	0.0246 (8)	−0.0013 (6)	0.0019 (6)	−0.0007 (7)
O46	0.0275 (8)	0.0276 (8)	0.0162 (8)	−0.0043 (7)	−0.0055 (6)	−0.0027 (6)
C47	0.0527 (17)	0.0408 (15)	0.0178 (12)	−0.0094 (12)	−0.0063 (11)	−0.0076 (11)
O48	0.0644 (14)	0.0747 (15)	0.0206 (9)	−0.0292 (12)	−0.0006 (9)	−0.0013 (9)
C49	0.088 (3)	0.059 (2)	0.0502 (19)	−0.0207 (19)	−0.0357 (18)	0.0223 (16)

### (±)-(1SR,5SR,6SR,7SR,10SR,11SR,13RS,14SR,15SR,16RS)-13-Acetoxy-16-benzyloxy-15-hydroxy-7-methoxymethoxy-3-oxo-11,15,18,18-tetramethyl-2,4-dioxatetracyclo[12.3.1.01,5.06,11]octadecan-10-yl benzoate (A) Geometric parameters (Å, º)

**Table:**

C1—O22	1.465 (2)	C18—H18C	0.9800
C1—C14	1.527 (3)	C19—H19A	0.9800
C1—C15	1.545 (3)	C19—H19B	0.9800
C1—C2	1.552 (3)	C19—H19C	0.9800
C2—O20	1.450 (2)	O20—C21	1.337 (3)
C2—C3	1.536 (3)	C21—O23	1.197 (2)
C2—H2	1.0000	C21—O22	1.327 (3)
C3—C4	1.533 (3)	O24—C25	1.347 (2)
C3—C8	1.556 (3)	C25—O26	1.199 (3)
C3—H3	1.0000	C25—C27	1.491 (3)
C4—O46	1.440 (2)	C27—C32	1.386 (3)
C4—C5	1.520 (3)	C27—C28	1.391 (3)
C4—H4	1.0000	C28—C29	1.379 (3)
C5—C6	1.526 (3)	C28—H28	0.9500
C5—H5A	0.9900	C29—C30	1.379 (4)
C5—H5B	0.9900	C29—H29	0.9500
C6—C7	1.512 (3)	C30—C31	1.381 (3)
C6—H6A	0.9900	C30—H30	0.9500
C6—H6B	0.9900	C31—C32	1.381 (3)
C7—O24	1.456 (2)	C31—H31	0.9500
C7—C8	1.547 (3)	C32—H32	0.9500
C7—H7	1.0000	O33—C34	1.344 (3)
C8—C19	1.536 (3)	C34—O35	1.211 (3)
C8—C9	1.551 (3)	C34—C36	1.498 (3)
C9—C10	1.548 (3)	C36—H36A	0.9800
C9—H9A	0.9900	C36—H36B	0.9800
C9—H9B	0.9900	C36—H36C	0.9800
C10—O33	1.468 (2)	O37—C38	1.435 (3)
C10—C11	1.564 (3)	C38—C39	1.508 (3)
C10—H10	1.0000	C38—H38A	0.9900
C11—C12	1.553 (3)	C38—H38B	0.9900
C11—C15	1.575 (3)	C39—C44	1.377 (3)
C11—H11	1.0000	C39—C40	1.393 (3)
C12—O45	1.437 (2)	C40—C41	1.379 (4)
C12—C13	1.528 (3)	C40—H40	0.9500
C12—C18	1.533 (3)	C41—C42	1.379 (4)
C13—O37	1.428 (2)	C41—H41	0.9500
C13—C14	1.535 (3)	C42—C43	1.370 (4)
C13—H13	1.0000	C42—H42	0.9500
C14—H14A	0.9900	C43—C44	1.391 (3)
C14—H14B	0.9900	C43—H43	0.9500
C15—C17	1.540 (3)	C44—H44	0.9500
C15—C16	1.549 (3)	O45—H45	0.8400
C16—H16A	0.9800	O46—C47	1.397 (3)
C16—H16B	0.9800	C47—O48	1.401 (3)
C16—H16C	0.9800	C47—H47A	0.9900
C17—H17A	0.9800	C47—H47B	0.9900
C17—H17B	0.9800	O48—C49	1.424 (4)
C17—H17C	0.9800	C49—H49A	0.9800
C18—H18A	0.9800	C49—H49B	0.9800
C18—H18B	0.9800	C49—H49C	0.9800
			
O22—C1—C14	104.90 (15)	C15—C17—H17A	109.5000
O22—C1—C15	108.22 (16)	C15—C17—H17B	109.5000
C14—C1—C15	111.99 (17)	H17A—C17—H17B	109.5000
O22—C1—C2	101.96 (15)	C15—C17—H17C	109.5000
C14—C1—C2	116.96 (17)	H17A—C17—H17C	109.5000
C15—C1—C2	111.72 (17)	H17B—C17—H17C	109.5000
O20—C2—C3	112.47 (16)	C12—C18—H18A	109.5000
O20—C2—C1	103.98 (15)	C12—C18—H18B	109.5000
C3—C2—C1	120.67 (17)	H18A—C18—H18B	109.5000
O20—C2—H2	106.3000	C12—C18—H18C	109.5000
C3—C2—H2	106.3000	H18A—C18—H18C	109.5000
C1—C2—H2	106.3000	H18B—C18—H18C	109.5000
C2—C3—C4	112.08 (16)	C8—C19—H19A	109.5000
C2—C3—C8	108.85 (16)	C8—C19—H19B	109.5000
C4—C3—C8	115.21 (16)	H19A—C19—H19B	109.5000
C2—C3—H3	106.7000	C8—C19—H19C	109.5000
C4—C3—H3	106.7000	H19A—C19—H19C	109.5000
C8—C3—H3	106.7000	H19B—C19—H19C	109.5000
O46—C4—C5	109.73 (17)	C21—O20—C2	110.54 (15)
O46—C4—C3	103.30 (16)	O23—C21—O22	124.5 (2)
C5—C4—C3	112.42 (16)	O23—C21—O20	123.5 (2)
O46—C4—H4	110.4000	O22—C21—O20	111.91 (17)
C5—C4—H4	110.4000	C21—O22—C1	111.51 (15)
C3—C4—H4	110.4000	C25—O24—C7	117.82 (16)
C4—C5—C6	112.91 (17)	O26—C25—O24	124.0 (2)
C4—C5—H5A	109.0000	O26—C25—C27	124.6 (2)
C6—C5—H5A	109.0000	O24—C25—C27	111.42 (18)
C4—C5—H5B	109.0000	C32—C27—C28	119.6 (2)
C6—C5—H5B	109.0000	C32—C27—C25	122.85 (19)
H5A—C5—H5B	107.8000	C28—C27—C25	117.5 (2)
C7—C6—C5	109.13 (17)	C29—C28—C27	120.2 (2)
C7—C6—H6A	109.9000	C29—C28—H28	119.9000
C5—C6—H6A	109.9000	C27—C28—H28	119.9000
C7—C6—H6B	109.9000	C30—C29—C28	119.9 (2)
C5—C6—H6B	109.9000	C30—C29—H29	120.0000
H6A—C6—H6B	108.3000	C28—C29—H29	120.0000
O24—C7—C6	110.06 (16)	C29—C30—C31	120.1 (2)
O24—C7—C8	106.44 (16)	C29—C30—H30	119.9000
C6—C7—C8	113.56 (17)	C31—C30—H30	119.9000
O24—C7—H7	108.9000	C32—C31—C30	120.3 (2)
C6—C7—H7	108.9000	C32—C31—H31	119.8000
C8—C7—H7	108.9000	C30—C31—H31	119.8000
C19—C8—C7	109.50 (16)	C31—C32—C27	119.8 (2)
C19—C8—C9	110.79 (16)	C31—C32—H32	120.1000
C7—C8—C9	104.79 (16)	C27—C32—H32	120.1000
C19—C8—C3	112.70 (16)	C34—O33—C10	118.86 (16)
C7—C8—C3	106.23 (16)	O35—C34—O33	124.5 (2)
C9—C8—C3	112.39 (16)	O35—C34—C36	126.0 (2)
C10—C9—C8	125.07 (16)	O33—C34—C36	109.49 (18)
C10—C9—H9A	106.1000	C34—C36—H36A	109.5000
C8—C9—H9A	106.1000	C34—C36—H36B	109.5000
C10—C9—H9B	106.1000	H36A—C36—H36B	109.5000
C8—C9—H9B	106.1000	C34—C36—H36C	109.5000
H9A—C9—H9B	106.3000	H36A—C36—H36C	109.5000
O33—C10—C9	100.10 (15)	H36B—C36—H36C	109.5000
O33—C10—C11	106.94 (15)	C13—O37—C38	114.54 (15)
C9—C10—C11	124.29 (17)	O37—C38—C39	113.51 (17)
O33—C10—H10	108.1000	O37—C38—H38A	108.9000
C9—C10—H10	108.1000	C39—C38—H38A	108.9000
C11—C10—H10	108.1000	O37—C38—H38B	108.9000
C12—C11—C10	114.77 (17)	C39—C38—H38B	108.9000
C12—C11—C15	113.35 (16)	H38A—C38—H38B	107.7000
C10—C11—C15	114.96 (16)	C44—C39—C40	118.6 (2)
C12—C11—H11	104.0000	C44—C39—C38	120.6 (2)
C10—C11—H11	104.0000	C40—C39—C38	120.8 (2)
C15—C11—H11	104.0000	C41—C40—C39	120.5 (2)
O45—C12—C13	109.17 (16)	C41—C40—H40	119.8000
O45—C12—C18	105.65 (16)	C39—C40—H40	119.8000
C13—C12—C18	110.59 (17)	C40—C41—C42	120.3 (2)
O45—C12—C11	105.79 (16)	C40—C41—H41	119.8000
C13—C12—C11	111.54 (16)	C42—C41—H41	119.8000
C18—C12—C11	113.74 (17)	C43—C42—C41	119.7 (2)
O37—C13—C12	105.82 (16)	C43—C42—H42	120.1000
O37—C13—C14	110.68 (16)	C41—C42—H42	120.1000
C12—C13—C14	114.47 (17)	C42—C43—C44	120.1 (3)
O37—C13—H13	108.6000	C42—C43—H43	120.0000
C12—C13—H13	108.6000	C44—C43—H43	120.0000
C14—C13—H13	108.6000	C39—C44—C43	120.7 (2)
C1—C14—C13	115.57 (17)	C39—C44—H44	119.6000
C1—C14—H14A	108.4000	C43—C44—H44	119.6000
C13—C14—H14A	108.4000	C12—O45—H45	109.5000
C1—C14—H14B	108.4000	C47—O46—C4	116.17 (17)
C13—C14—H14B	108.4000	O46—C47—O48	113.7 (2)
H14A—C14—H14B	107.4000	O46—C47—H47A	108.8000
C17—C15—C16	103.34 (17)	O48—C47—H47A	108.8000
C17—C15—C1	108.91 (17)	O46—C47—H47B	108.8000
C16—C15—C1	113.44 (17)	O48—C47—H47B	108.8000
C17—C15—C11	112.13 (17)	H47A—C47—H47B	107.7000
C16—C15—C11	110.84 (17)	C47—O48—C49	112.3 (2)
C1—C15—C11	108.17 (16)	O48—C49—H49A	109.5000
C15—C16—H16A	109.5000	O48—C49—H49B	109.5000
C15—C16—H16B	109.5000	H49A—C49—H49B	109.5000
H16A—C16—H16B	109.5000	O48—C49—H49C	109.5000
C15—C16—H16C	109.5000	H49A—C49—H49C	109.5000
H16A—C16—H16C	109.5000	H49B—C49—H49C	109.5000
H16B—C16—H16C	109.5000		
			
O22—C1—C2—O20	2.67 (19)	C2—C1—C15—C17	−159.97 (16)
C14—C1—C2—O20	−111.06 (18)	O22—C1—C15—C16	66.0 (2)
C15—C1—C2—O20	118.04 (17)	C14—C1—C15—C16	−178.89 (17)
O22—C1—C2—C3	129.94 (18)	C2—C1—C15—C16	−45.5 (2)
C14—C1—C2—C3	16.2 (3)	O22—C1—C15—C11	−170.62 (15)
C15—C1—C2—C3	−114.7 (2)	C14—C1—C15—C11	−55.5 (2)
O20—C2—C3—C4	1.6 (2)	C2—C1—C15—C11	77.9 (2)
C1—C2—C3—C4	−121.71 (19)	C12—C11—C15—C17	−62.1 (2)
O20—C2—C3—C8	−127.02 (17)	C10—C11—C15—C17	163.14 (18)
C1—C2—C3—C8	109.7 (2)	C12—C11—C15—C16	−177.01 (17)
C2—C3—C4—O46	67.6 (2)	C10—C11—C15—C16	48.2 (2)
C8—C3—C4—O46	−167.20 (16)	C12—C11—C15—C1	58.0 (2)
C2—C3—C4—C5	−174.17 (17)	C10—C11—C15—C1	−76.7 (2)
C8—C3—C4—C5	−49.0 (2)	C3—C2—O20—C21	−133.87 (18)
O46—C4—C5—C6	163.73 (16)	C1—C2—O20—C21	−1.7 (2)
C3—C4—C5—C6	49.4 (2)	C2—O20—C21—O23	−179.5 (2)
C4—C5—C6—C7	−55.0 (2)	C2—O20—C21—O22	−0.2 (2)
C5—C6—C7—O24	−179.23 (16)	O23—C21—O22—C1	−178.5 (2)
C5—C6—C7—C8	61.6 (2)	O20—C21—O22—C1	2.2 (2)
O24—C7—C8—C19	−57.7 (2)	C14—C1—O22—C21	119.40 (18)
C6—C7—C8—C19	63.6 (2)	C15—C1—O22—C21	−120.90 (18)
O24—C7—C8—C9	61.19 (19)	C2—C1—O22—C21	−3.0 (2)
C6—C7—C8—C9	−177.56 (16)	C6—C7—O24—C25	95.9 (2)
O24—C7—C8—C3	−179.65 (15)	C8—C7—O24—C25	−140.65 (18)
C6—C7—C8—C3	−58.4 (2)	C7—O24—C25—O26	−8.7 (3)
C2—C3—C8—C19	58.4 (2)	C7—O24—C25—C27	170.62 (17)
C4—C3—C8—C19	−68.5 (2)	O26—C25—C27—C32	169.0 (2)
C2—C3—C8—C7	178.28 (16)	O24—C25—C27—C32	−10.2 (3)
C4—C3—C8—C7	51.4 (2)	O26—C25—C27—C28	−7.5 (4)
C2—C3—C8—C9	−67.7 (2)	O24—C25—C27—C28	173.2 (2)
C4—C3—C8—C9	165.48 (17)	C32—C27—C28—C29	1.9 (4)
C19—C8—C9—C10	−60.1 (2)	C25—C27—C28—C29	178.6 (2)
C7—C8—C9—C10	−178.06 (18)	C27—C28—C29—C30	−1.1 (4)
C3—C8—C9—C10	67.0 (2)	C28—C29—C30—C31	−0.4 (4)
C8—C9—C10—O33	152.53 (18)	C29—C30—C31—C32	1.1 (4)
C8—C9—C10—C11	−88.8 (3)	C30—C31—C32—C27	−0.3 (4)
O33—C10—C11—C12	74.6 (2)	C28—C27—C32—C31	−1.2 (3)
C9—C10—C11—C12	−40.8 (3)	C25—C27—C32—C31	−177.7 (2)
O33—C10—C11—C15	−151.27 (16)	C9—C10—O33—C34	−150.15 (17)
C9—C10—C11—C15	93.3 (2)	C11—C10—O33—C34	79.1 (2)
C10—C11—C12—O45	−159.43 (15)	C10—O33—C34—O35	−1.6 (3)
C15—C11—C12—O45	65.7 (2)	C10—O33—C34—C36	177.18 (17)
C10—C11—C12—C13	82.0 (2)	C12—C13—O37—C38	163.72 (16)
C15—C11—C12—C13	−52.9 (2)	C14—C13—O37—C38	−71.7 (2)
C10—C11—C12—C18	−43.9 (2)	C13—O37—C38—C39	−59.1 (2)
C15—C11—C12—C18	−178.78 (17)	O37—C38—C39—C44	129.3 (2)
O45—C12—C13—O37	50.4 (2)	O37—C38—C39—C40	−51.7 (3)
C18—C12—C13—O37	−65.5 (2)	C44—C39—C40—C41	−0.2 (3)
C11—C12—C13—O37	166.91 (15)	C38—C39—C40—C41	−179.1 (2)
O45—C12—C13—C14	−71.8 (2)	C39—C40—C41—C42	1.2 (4)
C18—C12—C13—C14	172.39 (17)	C40—C41—C42—C43	−0.4 (4)
C11—C12—C13—C14	44.8 (2)	C41—C42—C43—C44	−1.6 (4)
O22—C1—C14—C13	167.95 (16)	C40—C39—C44—C43	−1.8 (3)
C15—C1—C14—C13	50.8 (2)	C38—C39—C44—C43	177.2 (2)
C2—C1—C14—C13	−80.0 (2)	C42—C43—C44—C39	2.6 (4)
O37—C13—C14—C1	−164.43 (17)	C5—C4—O46—C47	92.5 (2)
C12—C13—C14—C1	−45.0 (2)	C3—C4—O46—C47	−147.38 (18)
O22—C1—C15—C17	−48.5 (2)	C4—O46—C47—O48	−77.2 (3)
C14—C1—C15—C17	66.6 (2)	O46—C47—O48—C49	−63.1 (3)

### (±)-(1SR,5SR,6SR,7SR,10SR,11SR,13RS,14SR,15SR,16RS)-13-Acetoxy-16-benzyloxy-15-hydroxy-7-methoxymethoxy-3-oxo-11,15,18,18-tetramethyl-2,4-dioxatetracyclo[12.3.1.01,5.06,11]octadecan-10-yl benzoate (A) Hydrogen-bond geometry (Å, º)

**Table:**

*D*—H···*A*	*D*—H	H···*A*	*D*···*A*	*D*—H···*A*
C11—H11···O35	1.00	2.59	3.179 (2)	118
O45—H45···O35^i^	0.84	2.50	3.048 (2)	124
C38—H38*A*···O24^ii^	0.99	2.54	3.483 (3)	159
C19—H19*A*···O23^iii^	0.98	2.55	3.496 (3)	163
C29—H29···O33^iv^	0.95	2.57	3.510 (3)	173
C38—H38*B*···O23^v^	0.99	2.62	3.506 (3)	149
C6—H6*A*···O23^iii^	0.99	2.63	3.537 (3)	152

Symmetry codes: (i) −*x*, −*y*+1, −*z*+2; (ii) *x*−1, *y*, *z*; (iii) −*x*+1, −*y*+1, −*z*+1; (iv) −*x*+1, −*y*+2, −*z*+2; (v) −*x*, −*y*+1, −*z*+1.

### (±)-(1SR,5SR,6SR,7SR,10SR,11SR,13SR,14SR,15SR,16RS)-13-Acetoxy-16-benzyloxy-15-hydroxy-7-methoxymethoxy-3-oxo-11,15,18,18-tetramethyl-2,4-dioxatetracyclo[12.3.1.01,5.06,11]octadecan-10-yl benzoate (B) Crystal data

**Table:**

C_38_H_48_O_11_	*F*(000) = 728
*M**_r_* = 680.76	*D*_x_ = 1.335 Mg m^−^^3^
Triclinic, *P*1	Melting point = 476.5–478 K
*a* = 9.6913 (7) Å	Mo *K*α radiation, λ = 0.71073 Å
*b* = 11.8313 (8) Å	Cell parameters from 4246 reflections
*c* = 14.9295 (9) Å	θ = 2.3–24.7°
α = 96.304 (2)°	µ = 0.10 mm^−^^1^
β = 94.004 (2)°	*T* = 90 K
γ = 93.651 (2)°	Prism, colourless
*V* = 1692.9 (2) Å^3^	0.19 × 0.17 × 0.09 mm
*Z* = 2	

### (±)-(1SR,5SR,6SR,7SR,10SR,11SR,13SR,14SR,15SR,16RS)-13-Acetoxy-16-benzyloxy-15-hydroxy-7-methoxymethoxy-3-oxo-11,15,18,18-tetramethyl-2,4-dioxatetracyclo[12.3.1.01,5.06,11]octadecan-10-yl benzoate (B) Data collection

**Table:**

Bruker D8 Venture diffractometer	5809 independent reflections
Radiation source: fine-focus sealed tube	3577 reflections with *I* > 2σ(*I*)
Multilayered confocal mirror monochromator	*R*_int_ = 0.090
Detector resolution: 10.4167 pixels mm^-1^	θ_max_ = 25.0°, θ_min_ = 2.1°
φ and ω scans	*h* = −10→11
Absorption correction: multi-scan (SADABS; Krause *et al.*, 2015)	*k* = −14→12
*T*_min_ = 0.98, *T*_max_ = 0.99	*l* = −17→17
15750 measured reflections	

### (±)-(1SR,5SR,6SR,7SR,10SR,11SR,13SR,14SR,15SR,16RS)-13-Acetoxy-16-benzyloxy-15-hydroxy-7-methoxymethoxy-3-oxo-11,15,18,18-tetramethyl-2,4-dioxatetracyclo[12.3.1.01,5.06,11]octadecan-10-yl benzoate (B) Refinement

**Table:**

Refinement on *F*^2^	Primary atom site location: structure-invariant direct methods
Least-squares matrix: full	Secondary atom site location: difference Fourier map
*R*[*F*^2^ > 2σ(*F*^2^)] = 0.045	Hydrogen site location: inferred from neighbouring sites
*wR*(*F*^2^) = 0.096	H-atom parameters constrained
*S* = 1.03	*w* = 1/[σ^2^(*F*_o_^2^) + 0.6809*P*] where *P* = (*F*_o_^2^ + 2*F*_c_^2^)/3
5809 reflections	(Δ/σ)_max_ = 0.002
551 parameters	Δρ_max_ = 0.26 e Å^−^^3^
81 restraints	Δρ_min_ = −0.22 e Å^−^^3^

### (±)-(1SR,5SR,6SR,7SR,10SR,11SR,13SR,14SR,15SR,16RS)-13-Acetoxy-16-benzyloxy-15-hydroxy-7-methoxymethoxy-3-oxo-11,15,18,18-tetramethyl-2,4-dioxatetracyclo[12.3.1.01,5.06,11]octadecan-10-yl benzoate (B) Special details

**Table:**

Experimental. IR (film): 3545, 2947, 1800, 1738, 1721, 1271, 1099, 1041 cm^-1^; ^1^H NMR (500 MHz, CDCl_3_): *δ* (p.p.m.) 7.98–7.94 (m, 2H), 7.58–7.52 (m, 1H), 7.47–7.37 (m, 7H), 5.30–5.18 (m, 1H), 4.99–4.92 (m, 1H), 4.82 (d, *J* = 6.9 Hz, 1H), 4.78 (d, *J* = 12.0 Hz, 1H), 4.74–4.64 (m, 1H), 4.67 (d, *J* = 6.9 Hz, 1H), 4.60 (d, *J* = 12.0 Hz, 1H), 3.89 (dd, *J* = 9.7, 8.9 Hz, 1H), 3.68 (ddd, *J* = 10.3, 10.0, 4.9 Hz, 1H), 3.42 (s, 3H), 2.85–2.71 (m, 1H), 2.74 (brs, 1H), 2.36 (dddd, *J* = 13.5, 4.9, 4.0, 4.0 Hz, 1H), 2.33 (d, *J* = 4.3 Hz, 1H), 2.24–2.14 (m, 2H), 2.16–2.06 (m, 1H), 1.87–1.81 (m, 1H), 1.85 (s, 3H), 1.73–1.60 (m, 2H), 1.47 (s, 3H), 1.45–1.38 (m, 1H), 1.41 (s, 3H), 1.32 (s, 3H), 1.21 (s, 3H); HRMS (ESI): *m/z* calcd for C_38_H_48_O_11_Na^+^ [*M* + Na]^+^ 703.3094, found 703.3062.
Geometry. All esds (except the esd in the dihedral angle between two l.s. planes) are estimated using the full covariance matrix. The cell esds are taken into account individually in the estimation of esds in distances, angles and torsion angles; correlations between esds in cell parameters are only used when they are defined by crystal symmetry. An approximate (isotropic) treatment of cell esds is used for estimating esds involving l.s. planes.
Refinement. Refinement of *F*^2^ against ALL reflections. The weighted *R*-factor *wR* and goodness of fit *S* are based on *F*^2^, conventional *R*-factors *R* are based on *F*, with *F* set to zero for negative *F*^2^. The threshold expression of *F*^2^ > 2*σ*(*F*^2^) is used only for calculating *R*-factors(gt) *etc.* and is not relevant to the choice of reflections for refinement. *R*-factors based on *F*^2^ are statistically about twice as large as those based on *F*, and *R*-factors based on ALL data will be even larger. Model structure was improved by utilizing the BUMP command to solve the intramolecular short contact of 1.78 Å between atoms H2 and H16C, and the RIGU commands to estimate *U* values of the disordered benzene ring. Problematic 4 reflections with |I(obs)–I(calc)|/σW(I) greater than 10 (0 10 7, 2 6 11, 0 1 1 and 2 –1 1) have been omitted in the final refinement.

### (±)-(1SR,5SR,6SR,7SR,10SR,11SR,13SR,14SR,15SR,16RS)-13-Acetoxy-16-benzyloxy-15-hydroxy-7-methoxymethoxy-3-oxo-11,15,18,18-tetramethyl-2,4-dioxatetracyclo[12.3.1.01,5.06,11]octadecan-10-yl benzoate (B) Fractional atomic coordinates and isotropic or equivalent isotropic displacement parameters (Å^2^)

**Table:**

	*x*	*y*	*z*	*U*_iso_*/*U*_eq_	Occ. (<1)
C1	0.8584 (2)	1.01872 (17)	0.18691 (13)	0.0200 (5)	
C2	0.7051 (2)	0.97852 (17)	0.15839 (13)	0.0191 (5)	
H2	0.649435	1.011920	0.207354	0.023000*	
C3	0.6600 (2)	0.85028 (17)	0.14210 (13)	0.0178 (5)	
H3	0.745136	0.808839	0.153552	0.021000*	
C4	0.6051 (2)	0.81211 (18)	0.04417 (14)	0.0250 (6)	
H4	0.529296	0.859996	0.025251	0.030000*	
C5	0.5540 (3)	0.68564 (18)	0.03201 (15)	0.0323 (6)	
H5A	0.506930	0.666041	−0.029242	0.039000*	
H5B	0.634889	0.638951	0.036737	0.039000*	
C6	0.4549 (2)	0.65616 (19)	0.10148 (15)	0.0303 (6)	
H6A	0.368939	0.696009	0.093016	0.036000*	
H6B	0.429691	0.573058	0.093685	0.036000*	
C7	0.5233 (2)	0.69191 (18)	0.19519 (14)	0.0230 (5)	
H7	0.610172	0.651344	0.202360	0.028000*	
C8	0.5608 (2)	0.82212 (17)	0.21438 (14)	0.0189 (5)	
C9	0.6334 (2)	0.83985 (17)	0.31171 (13)	0.0209 (5)	
H9A	0.562886	0.812449	0.350731	0.025000*	
H9B	0.705526	0.784250	0.311368	0.025000*	
C10	0.7037 (2)	0.95049 (18)	0.36518 (14)	0.0273 (6)	
H10	0.711752	0.931768	0.428963	0.033000*	
C11	0.8497 (2)	1.00841 (18)	0.35329 (14)	0.0275 (6)	
H11	0.872348	1.058574	0.411532	0.033000*	
C12	0.9612 (2)	0.91953 (19)	0.35470 (14)	0.0272 (6)	
C13	0.9669 (2)	0.85450 (18)	0.26075 (14)	0.0213 (5)	
H13	0.885099	0.797483	0.249506	0.026000*	
C14	0.9653 (2)	0.92946 (18)	0.18310 (14)	0.0229 (5)	
H14A	1.058505	0.968900	0.183074	0.028000*	
H14B	0.947357	0.879446	0.125242	0.028000*	
C15	0.8702 (3)	1.09239 (18)	0.27930 (15)	0.0294 (6)	
C16	0.7663 (3)	1.18656 (18)	0.28164 (16)	0.0413 (7)	
H16A	0.785421	1.236535	0.235135	0.062000*	
H16B	0.776070	1.231434	0.341229	0.062000*	
H16C	0.671494	1.151321	0.270042	0.062000*	
C17	1.0129 (3)	1.1615 (2)	0.29506 (17)	0.0467 (8)	
H17A	1.086776	1.110351	0.282773	0.070000*	
H17B	1.026175	1.196576	0.357913	0.070000*	
H17C	1.016083	1.221111	0.254443	0.070000*	
C18	0.9425 (3)	0.83633 (19)	0.42508 (14)	0.0309 (6)	
H18A	1.026068	0.794578	0.431805	0.046000*	
H18B	0.862485	0.782296	0.405338	0.046000*	
H18C	0.926940	0.878766	0.483216	0.046000*	
C19	0.4281 (2)	0.88623 (18)	0.20956 (15)	0.0265 (6)	
H19A	0.382138	0.871255	0.148436	0.040000*	
H19B	0.451959	0.968247	0.224121	0.040000*	
H19C	0.365526	0.860099	0.253065	0.040000*	
O20	0.67221 (15)	1.03694 (11)	0.07988 (9)	0.0237 (4)	
C21	0.7839 (2)	1.09881 (18)	0.05953 (15)	0.0224 (5)	
O22	0.89293 (15)	1.09196 (12)	0.11742 (9)	0.0251 (4)	
O23	0.78395 (16)	1.15486 (12)	−0.00246 (10)	0.0309 (4)	
O24	0.43036 (15)	0.65942 (12)	0.26233 (10)	0.0267 (4)	
C25	0.4325 (5)	0.5480 (5)	0.2765 (4)	0.0212 (12)	0.499 (3)
O26	0.5034 (3)	0.4781 (3)	0.2418 (2)	0.0298 (10)	0.499 (3)
C27	0.3419 (5)	0.5281 (4)	0.3499 (4)	0.0186 (12)	0.499 (3)
C28	0.3138 (5)	0.4168 (4)	0.3681 (3)	0.0237 (11)	0.499 (3)
H28	0.348877	0.355343	0.332241	0.028000*	0.499 (3)
C29	0.2347 (5)	0.3960 (4)	0.4385 (3)	0.0261 (12)	0.499 (3)
H29	0.214081	0.319269	0.449194	0.031000*	0.499 (3)
C30	0.1847 (9)	0.4815 (6)	0.4935 (5)	0.0282 (18)	0.499 (3)
H30	0.133510	0.464943	0.543012	0.034000*	0.499 (3)
C31	0.2114 (14)	0.5973 (14)	0.4746 (12)	0.027 (3)	0.499 (3)
H31	0.175916	0.659433	0.509461	0.032000*	0.499 (3)
C32	0.2918 (14)	0.6139 (14)	0.4025 (11)	0.025 (3)	0.499 (3)
H32	0.312447	0.689838	0.389795	0.030000*	0.499 (3)
C25D	0.4773 (6)	0.5905 (4)	0.3285 (4)	0.0242 (12)	0.501 (3)
O26D	0.5882 (4)	0.5498 (3)	0.3245 (2)	0.0505 (12)	0.501 (3)
C27D	0.3793 (6)	0.5664 (5)	0.3954 (4)	0.0178 (12)	0.501 (3)
C28D	0.4112 (5)	0.4859 (4)	0.4534 (3)	0.0273 (12)	0.501 (3)
H28D	0.494422	0.448218	0.448256	0.033000*	0.501 (3)
C29D	0.3237 (5)	0.4600 (4)	0.5181 (3)	0.0302 (12)	0.501 (3)
H29D	0.345307	0.404208	0.557048	0.036000*	0.501 (3)
C30D	0.2042 (9)	0.5164 (6)	0.5256 (5)	0.033 (2)	0.501 (3)
H30D	0.143303	0.498983	0.570166	0.040000*	0.501 (3)
C31D	0.1725 (16)	0.5949 (15)	0.4714 (13)	0.032 (3)	0.501 (3)
H31D	0.087990	0.630649	0.477118	0.039000*	0.501 (3)
C32D	0.2592 (14)	0.6257 (13)	0.4072 (11)	0.017 (2)	0.501 (3)
H32D	0.238292	0.685344	0.371817	0.020000*	0.501 (3)
O33	0.59875 (19)	1.03335 (13)	0.36604 (10)	0.0378 (5)	
C34	0.5799 (3)	1.0968 (2)	0.44472 (18)	0.0382 (7)	
O35	0.64598 (19)	1.08938 (14)	0.51481 (11)	0.0415 (5)	
C36	0.4661 (3)	1.1731 (2)	0.42989 (19)	0.0600 (9)	
H36A	0.453922	1.220302	0.486550	0.090000*	
H36B	0.379770	1.126822	0.409696	0.090000*	
H36C	0.489674	1.222243	0.383663	0.090000*	
O37	1.08981 (15)	0.79399 (12)	0.26522 (10)	0.0274 (4)	
C38	1.1025 (2)	0.71208 (19)	0.18821 (15)	0.0287 (6)	
H38A	1.191912	0.676937	0.196142	0.034000*	
H38B	1.104522	0.752330	0.133529	0.034000*	
C39	0.9871 (2)	0.61915 (18)	0.17341 (14)	0.0218 (5)	
C40	0.9304 (3)	0.5816 (2)	0.08837 (16)	0.0408 (7)	
H40	0.960170	0.617578	0.038506	0.049000*	
C41	0.8296 (3)	0.4915 (2)	0.0738 (2)	0.0573 (9)	
H41	0.792518	0.465052	0.014086	0.069000*	
C42	0.7837 (3)	0.4409 (2)	0.1447 (2)	0.0426 (7)	
H42	0.714454	0.379419	0.134634	0.051000*	
C43	0.8375 (3)	0.4789 (2)	0.23040 (18)	0.0405 (7)	
H43	0.804578	0.444878	0.280332	0.049000*	
C44	0.9395 (3)	0.5665 (2)	0.24428 (16)	0.0359 (7)	
H44	0.978049	0.591329	0.304003	0.043000*	
O45	1.09175 (17)	0.98315 (13)	0.38187 (11)	0.0367 (5)	
H45	1.155296	0.938151	0.383312	0.055000*	
O46	0.72244 (18)	0.82882 (12)	−0.00707 (10)	0.0356 (4)	
C47	0.7298 (9)	0.8448 (7)	−0.0938 (6)	0.033 (2)	0.495 (4)
H47A	0.822280	0.881211	−0.101774	0.040000*	0.495 (4)
H47B	0.659356	0.897289	−0.110632	0.040000*	0.495 (4)
O48	0.7079 (4)	0.7415 (4)	−0.1521 (2)	0.0328 (11)	0.495 (4)
C49	0.8157 (5)	0.6683 (4)	−0.1399 (3)	0.0328 (15)	0.495 (4)
H49A	0.904539	0.713654	−0.127697	0.049000*	0.495 (4)
H49B	0.818482	0.615162	−0.194833	0.049000*	0.495 (4)
H49C	0.798756	0.625307	−0.088785	0.049000*	0.495 (4)
C47D	0.6639 (8)	0.8663 (7)	−0.0941 (6)	0.0284 (19)	0.505 (4)
H47D	0.613226	0.934996	−0.078710	0.034000*	0.505 (4)
H47E	0.742871	0.889946	−0.128149	0.034000*	0.505 (4)
O48D	0.5756 (4)	0.7894 (3)	−0.1522 (2)	0.0379 (13)	0.505 (4)
C49D	0.6421 (8)	0.6943 (6)	−0.1870 (4)	0.0491 (17)	0.505 (4)
H49D	0.717544	0.719139	−0.222313	0.074000*	0.505 (4)
H49E	0.575198	0.641821	−0.225880	0.074000*	0.505 (4)
H49F	0.679983	0.655508	−0.137022	0.074000*	0.505 (4)

### (±)-(1SR,5SR,6SR,7SR,10SR,11SR,13SR,14SR,15SR,16RS)-13-Acetoxy-16-benzyloxy-15-hydroxy-7-methoxymethoxy-3-oxo-11,15,18,18-tetramethyl-2,4-dioxatetracyclo[12.3.1.01,5.06,11]octadecan-10-yl benzoate (B) Atomic displacement parameters (Å^2^)

**Table:**

	*U*^11^	*U*^22^	*U*^33^	*U*^12^	*U*^13^	*U*^23^
C1	0.0262 (14)	0.0176 (12)	0.0165 (12)	−0.0034 (10)	−0.0001 (10)	0.0069 (10)
C2	0.0234 (13)	0.0188 (12)	0.0156 (11)	0.0026 (10)	0.0007 (9)	0.0044 (10)
C3	0.0183 (13)	0.0162 (12)	0.0185 (11)	0.0017 (10)	−0.0027 (9)	0.0021 (10)
C4	0.0336 (15)	0.0179 (12)	0.0218 (12)	−0.0007 (11)	−0.0078 (11)	0.0026 (10)
C5	0.0474 (17)	0.0196 (13)	0.0256 (13)	−0.0064 (12)	−0.0148 (12)	−0.0006 (11)
C6	0.0248 (15)	0.0188 (13)	0.0454 (15)	−0.0035 (11)	−0.0129 (12)	0.0079 (12)
C7	0.0178 (13)	0.0225 (13)	0.0301 (13)	0.0024 (10)	0.0037 (10)	0.0081 (11)
C8	0.0176 (13)	0.0162 (11)	0.0235 (12)	0.0011 (10)	0.0011 (10)	0.0048 (10)
C9	0.0263 (14)	0.0174 (12)	0.0198 (12)	0.0036 (11)	0.0039 (10)	0.0029 (10)
C10	0.0450 (17)	0.0200 (13)	0.0182 (12)	0.0110 (12)	0.0026 (11)	0.0028 (10)
C11	0.0477 (17)	0.0164 (12)	0.0153 (12)	−0.0028 (12)	−0.0068 (11)	−0.0033 (10)
C12	0.0315 (15)	0.0239 (13)	0.0238 (13)	−0.0074 (11)	−0.0120 (11)	0.0067 (11)
C13	0.0175 (13)	0.0219 (12)	0.0239 (13)	−0.0013 (10)	−0.0059 (10)	0.0058 (10)
C14	0.0201 (13)	0.0231 (12)	0.0248 (12)	−0.0030 (11)	−0.0045 (10)	0.0056 (10)
C15	0.0462 (17)	0.0187 (12)	0.0212 (13)	−0.0034 (12)	−0.0080 (11)	0.0022 (10)
C16	0.083 (2)	0.0175 (13)	0.0236 (14)	0.0086 (14)	−0.0003 (13)	0.0018 (11)
C17	0.072 (2)	0.0282 (14)	0.0345 (15)	−0.0247 (14)	−0.0250 (14)	0.0126 (12)
C18	0.0404 (16)	0.0258 (13)	0.0242 (13)	−0.0045 (12)	−0.0117 (11)	0.0055 (11)
C19	0.0236 (14)	0.0236 (13)	0.0344 (14)	0.0054 (11)	0.0064 (11)	0.0072 (11)
O20	0.0240 (9)	0.0200 (8)	0.0272 (9)	−0.0006 (7)	−0.0049 (7)	0.0089 (7)
C21	0.0259 (15)	0.0180 (12)	0.0217 (13)	−0.0005 (11)	−0.0030 (10)	−0.0001 (11)
O22	0.0260 (9)	0.0241 (8)	0.0249 (8)	−0.0042 (7)	−0.0055 (7)	0.0102 (7)
O23	0.0426 (11)	0.0266 (9)	0.0239 (9)	−0.0017 (8)	−0.0051 (7)	0.0124 (8)
O24	0.0209 (9)	0.0193 (9)	0.0426 (10)	0.0029 (7)	0.0046 (7)	0.0133 (8)
C25	0.017 (3)	0.019 (3)	0.027 (3)	−0.001 (3)	−0.004 (3)	0.005 (2)
O26	0.033 (2)	0.0181 (18)	0.041 (2)	0.0090 (17)	0.0152 (17)	0.0058 (16)
C27	0.020 (3)	0.018 (3)	0.017 (3)	0.002 (2)	−0.003 (2)	0.001 (2)
C28	0.030 (3)	0.018 (2)	0.024 (2)	0.004 (2)	0.000 (2)	0.0037 (19)
C29	0.026 (3)	0.024 (2)	0.028 (3)	−0.002 (2)	−0.001 (2)	0.009 (2)
C30	0.023 (4)	0.030 (4)	0.032 (4)	−0.003 (3)	0.003 (3)	0.007 (3)
C31	0.033 (7)	0.028 (4)	0.019 (4)	0.005 (4)	0.001 (4)	0.003 (3)
C32	0.029 (7)	0.023 (4)	0.023 (4)	0.004 (4)	0.002 (4)	0.005 (3)
C25D	0.022 (3)	0.026 (3)	0.025 (3)	0.005 (3)	−0.005 (3)	0.009 (3)
O26D	0.040 (3)	0.072 (3)	0.054 (3)	0.032 (2)	0.0210 (19)	0.045 (2)
C27D	0.019 (3)	0.014 (3)	0.022 (3)	0.002 (2)	0.002 (2)	0.003 (2)
C28D	0.023 (3)	0.029 (3)	0.033 (3)	0.008 (2)	0.002 (2)	0.012 (2)
C29D	0.030 (3)	0.031 (3)	0.032 (3)	0.001 (2)	0.002 (2)	0.015 (2)
C30D	0.026 (4)	0.036 (4)	0.039 (5)	−0.003 (3)	0.004 (3)	0.009 (3)
C31D	0.027 (6)	0.038 (4)	0.033 (4)	0.003 (4)	0.006 (5)	0.006 (3)
C32D	0.020 (5)	0.013 (4)	0.017 (4)	0.007 (3)	0.000 (3)	−0.001 (3)
O33	0.0673 (13)	0.0256 (9)	0.0248 (9)	0.0221 (9)	0.0135 (8)	0.0045 (8)
C34	0.068 (2)	0.0183 (14)	0.0307 (16)	0.0019 (14)	0.0247 (15)	0.0024 (13)
O35	0.0589 (13)	0.0348 (10)	0.0277 (10)	−0.0041 (9)	0.0099 (9)	−0.0103 (9)
C36	0.109 (3)	0.0329 (16)	0.0490 (18)	0.0363 (18)	0.0385 (18)	0.0138 (14)
O37	0.0211 (9)	0.0257 (9)	0.0347 (10)	−0.0004 (7)	−0.0090 (7)	0.0082 (8)
C38	0.0229 (14)	0.0333 (14)	0.0322 (14)	0.0073 (12)	0.0032 (11)	0.0097 (12)
C39	0.0195 (13)	0.0238 (13)	0.0227 (13)	0.0092 (11)	0.0011 (10)	0.0013 (11)
C40	0.072 (2)	0.0268 (14)	0.0223 (14)	0.0059 (15)	−0.0022 (13)	0.0004 (12)
C41	0.094 (3)	0.0254 (15)	0.0450 (18)	0.0006 (17)	−0.0371 (17)	−0.0025 (14)
C42	0.0332 (17)	0.0187 (14)	0.071 (2)	0.0036 (12)	−0.0138 (15)	−0.0082 (15)
C43	0.0487 (18)	0.0266 (14)	0.0435 (17)	−0.0095 (14)	0.0171 (14)	−0.0101 (13)
C44	0.0462 (18)	0.0348 (15)	0.0224 (13)	−0.0143 (13)	0.0005 (12)	−0.0043 (12)
O45	0.0414 (11)	0.0298 (9)	0.0343 (10)	−0.0153 (8)	−0.0208 (9)	0.0079 (8)
O46	0.0634 (13)	0.0254 (9)	0.0194 (9)	0.0025 (9)	0.0157 (8)	0.0020 (7)
C47	0.052 (6)	0.028 (4)	0.017 (3)	0.004 (4)	−0.004 (4)	−0.008 (3)
O48	0.034 (3)	0.042 (3)	0.021 (2)	0.004 (2)	−0.0027 (17)	−0.003 (2)
C49	0.039 (4)	0.029 (3)	0.030 (3)	0.010 (3)	0.008 (2)	−0.002 (2)
C47D	0.047 (5)	0.024 (4)	0.013 (3)	−0.008 (4)	−0.004 (4)	0.008 (3)
O48D	0.041 (3)	0.048 (3)	0.0209 (19)	−0.0078 (19)	−0.0031 (16)	−0.0006 (18)
C49D	0.053 (5)	0.057 (5)	0.036 (4)	−0.002 (4)	0.016 (3)	−0.007 (3)

### (±)-(1SR,5SR,6SR,7SR,10SR,11SR,13SR,14SR,15SR,16RS)-13-Acetoxy-16-benzyloxy-15-hydroxy-7-methoxymethoxy-3-oxo-11,15,18,18-tetramethyl-2,4-dioxatetracyclo[12.3.1.01,5.06,11]octadecan-10-yl benzoate (B) Geometric parameters (Å, º)

**Table:**

C1—O22	1.466 (2)	C27—C32	1.351 (14)
C1—C14	1.525 (3)	C27—C28	1.388 (6)
C1—C15	1.542 (3)	C28—C29	1.378 (6)
C1—C2	1.547 (3)	C28—H28	0.9500
C2—O20	1.452 (2)	C29—C30	1.367 (9)
C2—C3	1.540 (3)	C29—H29	0.9500
C2—H2	1.0000	C30—C31	1.440 (18)
C3—C4	1.530 (3)	C30—H30	0.9500
C3—C8	1.544 (3)	C31—C32	1.395 (17)
C3—H3	1.0000	C31—H31	0.9500
C4—O46	1.429 (3)	C32—H32	0.9500
C4—C5	1.533 (3)	C25D—O26D	1.208 (5)
C4—H4	1.0000	C25D—C27D	1.464 (7)
C5—C6	1.515 (3)	C27D—C28D	1.391 (6)
C5—H5A	0.9900	C27D—C32D	1.408 (12)
C5—H5B	0.9900	C28D—C29D	1.376 (6)
C6—C7	1.510 (3)	C28D—H28D	0.9500
C6—H6A	0.9900	C29D—C30D	1.377 (10)
C6—H6B	0.9900	C29D—H29D	0.9500
C7—O24	1.460 (2)	C30D—C31D	1.33 (2)
C7—C8	1.551 (3)	C30D—H30D	0.9500
C7—H7	1.0000	C31D—C32D	1.381 (17)
C8—C19	1.536 (3)	C31D—H31D	0.9500
C8—C9	1.556 (3)	C32D—H32D	0.9500
C9—C10	1.548 (3)	O33—C34	1.353 (3)
C9—H9A	0.9900	C34—O35	1.203 (3)
C9—H9B	0.9900	C34—C36	1.489 (4)
C10—O33	1.457 (3)	C36—H36A	0.9800
C10—C11	1.563 (3)	C36—H36B	0.9800
C10—H10	1.0000	C36—H36C	0.9800
C11—C12	1.556 (3)	O37—C38	1.437 (3)
C11—C15	1.579 (3)	C38—C39	1.505 (3)
C11—H11	1.0000	C38—H38A	0.9900
C12—O45	1.439 (3)	C38—H38B	0.9900
C12—C18	1.529 (3)	C39—C40	1.364 (3)
C12—C13	1.530 (3)	C39—C44	1.377 (3)
C13—O37	1.430 (2)	C40—C41	1.387 (4)
C13—C14	1.535 (3)	C40—H40	0.9500
C13—H13	1.0000	C41—C42	1.359 (4)
C14—H14A	0.9900	C41—H41	0.9500
C14—H14B	0.9900	C42—C43	1.364 (3)
C15—C16	1.548 (3)	C42—H42	0.9500
C15—C17	1.551 (3)	C43—C44	1.375 (3)
C16—H16A	0.9800	C43—H43	0.9500
C16—H16B	0.9800	C44—H44	0.9500
C16—H16C	0.9800	O45—H45	0.8400
C17—H17A	0.9800	O46—C47	1.334 (9)
C17—H17B	0.9800	O46—C47D	1.505 (7)
C17—H17C	0.9800	C47—O48	1.415 (10)
C18—H18A	0.9800	C47—H47A	0.9900
C18—H18B	0.9800	C47—H47B	0.9900
C18—H18C	0.9800	O48—C49	1.413 (6)
C19—H19A	0.9800	C49—H49A	0.9800
C19—H19B	0.9800	C49—H49B	0.9800
C19—H19C	0.9800	C49—H49C	0.9800
O20—C21	1.339 (3)	C47D—O48D	1.391 (9)
C21—O23	1.196 (2)	C47D—H47D	0.9900
C21—O22	1.330 (2)	C47D—H47E	0.9900
O24—C25	1.359 (5)	O48D—C49D	1.401 (7)
O24—C25D	1.418 (5)	C49D—H49D	0.9800
C25—O26	1.205 (5)	C49D—H49E	0.9800
C25—C27	1.480 (7)	C49D—H49F	0.9800
			
O22—C1—C14	105.13 (16)	C21—O20—C2	110.58 (16)
O22—C1—C15	108.54 (16)	O23—C21—O22	124.5 (2)
C14—C1—C15	111.58 (18)	O23—C21—O20	123.6 (2)
O22—C1—C2	102.40 (15)	O22—C21—O20	111.86 (18)
C14—C1—C2	118.03 (17)	C21—O22—C1	111.22 (16)
C15—C1—C2	110.25 (18)	C25—O24—C7	113.5 (3)
O20—C2—C3	112.46 (16)	C25D—O24—C7	119.5 (2)
O20—C2—C1	103.90 (15)	O26—C25—O24	127.1 (4)
C3—C2—C1	120.11 (18)	O26—C25—C27	124.6 (4)
O20—C2—H2	106.5000	O24—C25—C27	108.1 (4)
C3—C2—H2	106.5000	C32—C27—C28	118.9 (8)
C1—C2—H2	106.5000	C32—C27—C25	122.8 (8)
C4—C3—C2	112.45 (16)	C28—C27—C25	118.2 (5)
C4—C3—C8	115.39 (17)	C29—C28—C27	119.6 (4)
C2—C3—C8	108.34 (17)	C29—C28—H28	120.2000
C4—C3—H3	106.7000	C27—C28—H28	120.2000
C2—C3—H3	106.7000	C30—C29—C28	122.5 (5)
C8—C3—H3	106.7000	C30—C29—H29	118.8000
O46—C4—C3	104.44 (17)	C28—C29—H29	118.8000
O46—C4—C5	109.71 (19)	C29—C30—C31	118.5 (9)
C3—C4—C5	110.80 (17)	C29—C30—H30	120.8000
O46—C4—H4	110.6000	C31—C30—H30	120.8000
C3—C4—H4	110.6000	C32—C31—C30	116.8 (14)
C5—C4—H4	110.6000	C32—C31—H31	121.6000
C6—C5—C4	112.6 (2)	C30—C31—H31	121.6000
C6—C5—H5A	109.1000	C27—C32—C31	123.7 (14)
C4—C5—H5A	109.1000	C27—C32—H32	118.2000
C6—C5—H5B	109.1000	C31—C32—H32	118.2000
C4—C5—H5B	109.1000	O26D—C25D—O24	120.4 (4)
H5A—C5—H5B	107.8000	O26D—C25D—C27D	123.7 (4)
C7—C6—C5	109.32 (18)	O24—C25D—C27D	115.7 (4)
C7—C6—H6A	109.8000	C28D—C27D—C32D	118.8 (8)
C5—C6—H6A	109.8000	C28D—C27D—C25D	118.1 (5)
C7—C6—H6B	109.8000	C32D—C27D—C25D	123.1 (8)
C5—C6—H6B	109.8000	C29D—C28D—C27D	120.9 (5)
H6A—C6—H6B	108.3000	C29D—C28D—H28D	119.6000
O24—C7—C6	109.50 (17)	C27D—C28D—H28D	119.6000
O24—C7—C8	108.01 (17)	C28D—C29D—C30D	118.9 (5)
C6—C7—C8	113.13 (17)	C28D—C29D—H29D	120.5000
O24—C7—H7	108.7000	C30D—C29D—H29D	120.5000
C6—C7—H7	108.7000	C31D—C30D—C29D	121.2 (10)
C8—C7—H7	108.7000	C31D—C30D—H30D	119.4000
C19—C8—C3	112.98 (16)	C29D—C30D—H30D	119.4000
C19—C8—C7	109.77 (17)	C30D—C31D—C32D	121.8 (14)
C3—C8—C7	105.29 (17)	C30D—C31D—H31D	119.1000
C19—C8—C9	110.89 (17)	C32D—C31D—H31D	119.1000
C3—C8—C9	112.59 (17)	C31D—C32D—C27D	118.3 (13)
C7—C8—C9	104.83 (16)	C31D—C32D—H32D	120.8000
C10—C9—C8	128.84 (17)	C27D—C32D—H32D	120.8000
C10—C9—H9A	105.1000	C34—O33—C10	118.91 (19)
C8—C9—H9A	105.1000	O35—C34—O33	123.6 (2)
C10—C9—H9B	105.1000	O35—C34—C36	126.9 (2)
C8—C9—H9B	105.1000	O33—C34—C36	109.5 (2)
H9A—C9—H9B	105.9000	C34—C36—H36A	109.5000
O33—C10—C9	105.72 (18)	C34—C36—H36B	109.5000
O33—C10—C11	110.82 (17)	H36A—C36—H36B	109.5000
C9—C10—C11	127.33 (19)	C34—C36—H36C	109.5000
O33—C10—H10	103.4000	H36A—C36—H36C	109.5000
C9—C10—H10	103.4000	H36B—C36—H36C	109.5000
C11—C10—H10	103.4000	C13—O37—C38	114.57 (16)
C12—C11—C10	110.26 (17)	O37—C38—C39	113.76 (18)
C12—C11—C15	112.88 (19)	O37—C38—H38A	108.8000
C10—C11—C15	121.09 (19)	C39—C38—H38A	108.8000
C12—C11—H11	103.4000	O37—C38—H38B	108.8000
C10—C11—H11	103.4000	C39—C38—H38B	108.8000
C15—C11—H11	103.4000	H38A—C38—H38B	107.7000
O45—C12—C18	105.47 (17)	C40—C39—C44	118.1 (2)
O45—C12—C13	109.83 (18)	C40—C39—C38	120.5 (2)
C18—C12—C13	110.43 (18)	C44—C39—C38	121.3 (2)
O45—C12—C11	106.34 (17)	C39—C40—C41	120.7 (2)
C18—C12—C11	113.83 (19)	C39—C40—H40	119.6000
C13—C12—C11	110.69 (17)	C41—C40—H40	119.6000
O37—C13—C12	105.79 (17)	C42—C41—C40	120.3 (2)
O37—C13—C14	111.37 (17)	C42—C41—H41	119.9000
C12—C13—C14	114.68 (18)	C40—C41—H41	119.9000
O37—C13—H13	108.3000	C41—C42—C43	119.7 (2)
C12—C13—H13	108.3000	C41—C42—H42	120.1000
C14—C13—H13	108.3000	C43—C42—H42	120.1000
C1—C14—C13	115.06 (18)	C42—C43—C44	119.8 (2)
C1—C14—H14A	108.5000	C42—C43—H43	120.1000
C13—C14—H14A	108.5000	C44—C43—H43	120.1000
C1—C14—H14B	108.5000	C43—C44—C39	121.4 (2)
C13—C14—H14B	108.5000	C43—C44—H44	119.3000
H14A—C14—H14B	107.5000	C39—C44—H44	119.3000
C1—C15—C16	111.57 (18)	C12—O45—H45	109.5000
C1—C15—C17	110.3 (2)	C47—O46—C4	130.7 (4)
C16—C15—C17	102.98 (19)	C4—O46—C47D	104.8 (3)
C1—C15—C11	106.92 (16)	O46—C47—O48	112.4 (6)
C16—C15—C11	113.4 (2)	O46—C47—H47A	109.1000
C17—C15—C11	111.71 (18)	O48—C47—H47A	109.1000
C15—C16—H16A	109.5000	O46—C47—H47B	109.1000
C15—C16—H16B	109.5000	O48—C47—H47B	109.1000
H16A—C16—H16B	109.5000	H47A—C47—H47B	107.9000
C15—C16—H16C	109.5000	C49—O48—C47	112.2 (4)
H16A—C16—H16C	109.5000	O48—C49—H49A	109.5000
H16B—C16—H16C	109.5000	O48—C49—H49B	109.5000
C15—C17—H17A	109.5000	H49A—C49—H49B	109.5000
C15—C17—H17B	109.5000	O48—C49—H49C	109.5000
H17A—C17—H17B	109.5000	H49A—C49—H49C	109.5000
C15—C17—H17C	109.5000	H49B—C49—H49C	109.5000
H17A—C17—H17C	109.5000	O48D—C47D—O46	118.6 (6)
H17B—C17—H17C	109.5000	O48D—C47D—H47D	107.7000
C12—C18—H18A	109.5000	O46—C47D—H47D	107.7000
C12—C18—H18B	109.5000	O48D—C47D—H47E	107.7000
H18A—C18—H18B	109.5000	O46—C47D—H47E	107.7000
C12—C18—H18C	109.5000	H47D—C47D—H47E	107.1000
H18A—C18—H18C	109.5000	C47D—O48D—C49D	112.5 (6)
H18B—C18—H18C	109.5000	O48D—C49D—H49D	109.5000
C8—C19—H19A	109.5000	O48D—C49D—H49E	109.5000
C8—C19—H19B	109.5000	H49D—C49D—H49E	109.5000
H19A—C19—H19B	109.5000	O48D—C49D—H49F	109.5000
C8—C19—H19C	109.5000	H49D—C49D—H49F	109.5000
H19A—C19—H19C	109.5000	H49E—C49D—H49F	109.5000
H19B—C19—H19C	109.5000		
			
O22—C1—C2—O20	−2.10 (19)	C10—C11—C15—C16	50.2 (3)
C14—C1—C2—O20	−116.91 (18)	C12—C11—C15—C17	−60.1 (2)
C15—C1—C2—O20	113.25 (17)	C10—C11—C15—C17	166.0 (2)
O22—C1—C2—C3	124.67 (18)	C3—C2—O20—C21	−129.25 (18)
C14—C1—C2—C3	9.9 (3)	C1—C2—O20—C21	2.2 (2)
C15—C1—C2—C3	−120.0 (2)	C2—O20—C21—O23	179.9 (2)
O20—C2—C3—C4	6.0 (3)	C2—O20—C21—O22	−1.3 (2)
C1—C2—C3—C4	−116.7 (2)	O23—C21—O22—C1	178.5 (2)
O20—C2—C3—C8	−122.75 (18)	O20—C21—O22—C1	−0.2 (2)
C1—C2—C3—C8	114.5 (2)	C14—C1—O22—C21	125.39 (18)
C2—C3—C4—O46	65.1 (2)	C15—C1—O22—C21	−115.1 (2)
C8—C3—C4—O46	−169.98 (16)	C2—C1—O22—C21	1.5 (2)
C2—C3—C4—C5	−176.87 (19)	C6—C7—O24—C25	80.8 (3)
C8—C3—C4—C5	−51.9 (3)	C8—C7—O24—C25	−155.6 (3)
O46—C4—C5—C6	165.47 (17)	C6—C7—O24—C25D	124.7 (3)
C3—C4—C5—C6	50.7 (3)	C8—C7—O24—C25D	−111.7 (3)
C4—C5—C6—C7	−55.6 (2)	C7—O24—C25—O26	1.9 (7)
C5—C6—C7—O24	−177.67 (17)	C7—O24—C25—C27	176.2 (3)
C5—C6—C7—C8	61.8 (2)	O26—C25—C27—C32	162.7 (10)
C4—C3—C8—C19	−65.4 (2)	O24—C25—C27—C32	−11.8 (11)
C2—C3—C8—C19	61.7 (2)	O26—C25—C27—C28	−13.7 (8)
C4—C3—C8—C7	54.4 (2)	O24—C25—C27—C28	171.8 (4)
C2—C3—C8—C7	−178.56 (16)	C32—C27—C28—C29	0.6 (11)
C4—C3—C8—C9	168.03 (17)	C25—C27—C28—C29	177.2 (4)
C2—C3—C8—C9	−64.9 (2)	C27—C28—C29—C30	−1.7 (8)
O24—C7—C8—C19	−58.7 (2)	C28—C29—C30—C31	2.5 (12)
C6—C7—C8—C19	62.6 (2)	C29—C30—C31—C32	−2.3 (17)
O24—C7—C8—C3	179.36 (15)	C28—C27—C32—C31	−0.6 (18)
C6—C7—C8—C3	−59.3 (2)	C25—C27—C32—C31	−177.0 (11)
O24—C7—C8—C9	60.4 (2)	C30—C31—C32—C27	1 (2)
C6—C7—C8—C9	−178.23 (18)	C7—O24—C25D—O26D	−7.1 (7)
C19—C8—C9—C10	−66.7 (3)	C7—O24—C25D—C27D	177.4 (4)
C3—C8—C9—C10	61.0 (3)	O26D—C25D—C27D—C28D	−4.8 (9)
C7—C8—C9—C10	174.9 (2)	O24—C25D—C27D—C28D	170.5 (4)
C8—C9—C10—O33	55.6 (3)	O26D—C25D—C27D—C32D	171.3 (10)
C8—C9—C10—C11	−77.2 (3)	O24—C25D—C27D—C32D	−13.4 (11)
O33—C10—C11—C12	178.71 (16)	C32D—C27D—C28D—C29D	3.3 (11)
C9—C10—C11—C12	−50.4 (3)	C25D—C27D—C28D—C29D	179.6 (5)
O33—C10—C11—C15	−46.3 (3)	C27D—C28D—C29D—C30D	−0.8 (8)
C9—C10—C11—C15	84.6 (3)	C28D—C29D—C30D—C31D	0.0 (13)
C10—C11—C12—O45	−156.12 (16)	C29D—C30D—C31D—C32D	−2 (2)
C15—C11—C12—O45	65.0 (2)	C30D—C31D—C32D—C27D	4 (2)
C10—C11—C12—C18	−40.4 (2)	C28D—C27D—C32D—C31D	−4.9 (18)
C15—C11—C12—C18	−179.31 (17)	C25D—C27D—C32D—C31D	179.0 (11)
C10—C11—C12—C13	84.6 (2)	C9—C10—O33—C34	133.93 (19)
C15—C11—C12—C13	−54.2 (2)	C11—C10—O33—C34	−84.7 (2)
O45—C12—C13—O37	51.0 (2)	C10—O33—C34—O35	0.5 (3)
C18—C12—C13—O37	−64.9 (2)	C10—O33—C34—C36	−178.7 (2)
C11—C12—C13—O37	168.11 (16)	C12—C13—O37—C38	169.98 (16)
O45—C12—C13—C14	−72.1 (2)	C14—C13—O37—C38	−64.8 (2)
C18—C12—C13—C14	171.95 (19)	C13—O37—C38—C39	−60.8 (2)
C11—C12—C13—C14	45.0 (2)	O37—C38—C39—C40	139.0 (2)
O22—C1—C14—C13	169.91 (16)	O37—C38—C39—C44	−43.6 (3)
C15—C1—C14—C13	52.4 (2)	C44—C39—C40—C41	−1.2 (4)
C2—C1—C14—C13	−76.8 (2)	C38—C39—C40—C41	176.2 (2)
O37—C13—C14—C1	−165.42 (16)	C39—C40—C41—C42	1.5 (4)
C12—C13—C14—C1	−45.3 (3)	C40—C41—C42—C43	−0.2 (4)
O22—C1—C15—C16	62.0 (2)	C41—C42—C43—C44	−1.2 (4)
C14—C1—C15—C16	177.37 (18)	C42—C43—C44—C39	1.5 (4)
C2—C1—C15—C16	−49.4 (2)	C40—C39—C44—C43	−0.2 (4)
O22—C1—C15—C17	−51.8 (2)	C38—C39—C44—C43	−177.7 (2)
C14—C1—C15—C17	63.6 (2)	C3—C4—O46—C47	−156.2 (5)
C2—C1—C15—C17	−163.23 (17)	C5—C4—O46—C47	85.0 (5)
O22—C1—C15—C11	−173.48 (17)	C3—C4—O46—C47D	−144.5 (4)
C14—C1—C15—C11	−58.1 (2)	C5—C4—O46—C47D	96.7 (4)
C2—C1—C15—C11	75.1 (2)	C4—O46—C47—O48	−79.7 (6)
C12—C11—C15—C1	60.7 (2)	O46—C47—O48—C49	−66.8 (7)
C10—C11—C15—C1	−73.2 (2)	C4—O46—C47D—O48D	−65.8 (7)
C12—C11—C15—C16	−175.92 (18)	O46—C47D—O48D—C49D	−64.7 (7)

### (±)-(1SR,5SR,6SR,7SR,10SR,11SR,13SR,14SR,15SR,16RS)-13-Acetoxy-16-benzyloxy-15-hydroxy-7-methoxymethoxy-3-oxo-11,15,18,18-tetramethyl-2,4-dioxatetracyclo[12.3.1.01,5.06,11]octadecan-10-yl benzoate (B) Hydrogen-bond geometry (Å, º)

*Cg*2 is the centroid of the C27–C32 benzene ring.

**Table:**

*D*—H···*A*	*D*—H	H···*A*	*D*···*A*	*D*—H···*A*
C2—H2···O33	1.00	2.44	3.344 (3)	150
O45—H45···O35^i^	0.84	2.43	3.098 (2)	137
C18—H18*C*···O45^i^	0.98	2.47	3.442 (3)	169
C38—H38*A*···O24^ii^	0.99	2.48	3.409 (3)	156
C32*D*—H32*D*···O37^iii^	0.95	2.57	3.465 (16)	157
C19—H19*A*···O23^iv^	0.98	2.60	3.560 (3)	168
C16—H16*C*···O33	0.98	2.23	2.814 (3)	117
C47*D*—H47*D*···O20	0.99	2.55	3.103 (9)	115
C5—H5*A*···O48*D*	0.99	2.56	3.147 (4)	118
C38—H38*B*···O23^v^	0.99	2.61	3.542 (3)	157
C28*D*—H28*D*···*Cg*2^vi^	0.95	2.93	3.546 (7)	124

Symmetry codes: (i) −*x*+2, −*y*+2, −*z*+1; (ii) *x*+1, *y*, *z*; (iii) *x*−1, *y*, *z*; (iv) −*x*+1, −*y*+2, −*z*; (v) −*x*+2, −*y*+2, −*z*; (vi) −*x*+1, −*y*+1, −*z*+1.
